# Melt burst events in a dominantly amagmatic lithosphere at Gakkel Ridge in the Arctic Ocean

**DOI:** 10.1038/s41467-026-76409-3

**Published:** 2026-08-07

**Authors:** Fei Zhou, Ingo Grevemeyer, Jérôme Dyment

**Affiliations:** 1 GEOMAR Helmholtz Centre for Ocean Research Kiel, Kiel, Germany; 2 Université Paris Cité, Institut de Physique du Globe de Paris, CNRS, Paris, France

**Keywords:** Geophysics, Geomagnetism, Geodynamics, Tectonics

## Abstract

Alternation of magmatic and amagmatic seafloor along the slow and ultraslow spreading ridges is common. Yet, temporal variation is obscured by sediments blanketing oceanic basement away from spreading centers. Here we analyze aeromagnetic data covering zero age to ~35 Ma oceanic crust at the ultraslow-spreading Gakkel Ridge to characterize magmatic and amagmatic lithospheric domains and their links to melt supply. Amagmatic seafloor unexpectedly dominates ~68% of the Eurasian Basin, while only 32% of magmatic seafloor over the last 35 Ma. The melt supply exhibits cycles initiating over a short episode of robust magmatism followed by a longer intermediate magmatism, then starved magmatism. These observations suggest that strong lithosphere presumably characterizing this ultraslow spreading ridge may prevent a continuous melt supply, instead favoring episodic melt bursts. Our study indirectly constraints the extent and duration of melting events and lithospheric accretion through the Gakkel Ridge history, away from the present spreading axis.

## Abstract (web summary)

Aeromagnetic data reveal episodic melt bursts within a dominantly amagmatic lithosphere beneath the ultraslow-spreading Gakkel Ridge, suggesting that strong lithosphere may promote pulsed rather than continuous magma supply.

## Introduction

At slow and ultraslow spreading centers, low amounts of melting and magmatism induce a complex and laterally varying lithospheric structure^1,2^. Segments with thick crust reflect robust, focused magmatism and hence the occurrence of long-lived magma chambers. Segments dominated by tectonics may expose mantle rocks, hence reflecting starved magmatism^3–8^ lacking stable magma chambers. The along-axis segmentation of magmatic and amagmatic seafloor has been revealed by numerous observations at ultraslow spreading ridges^9–12^, allowing us to unravel the mechanism of melt supply related to the ridge axis segmentation^13,14^. In contrast to the along-axis studies, the nature of the lithosphere and the temporal variation of magmatism across the ridge axis are still unclear. Only a handful of seismic studies based on active source surveys revealed the occurrence of episodic magmatic and amagmatic seafloor and hence temporal variations of magmatic accretion across the Mid-Cayman Spreading Center and the Gakkel Ridge^15,16^. However, the observations along a few seismic profiles are clearly insufficient to depict the nature of the lithosphere and the melt supply from a regional perspective.

Here, we use aeromagnetic data over the Eurasian Basin, Arctic Ocean, to characterize the lithosphere and reveal the corresponding mechanism of melt supply. Marine magnetic data have previously been used to reveal and classify distinct domains of magmatic and amagmatic lithosphere at ultraslow spreading ridges^17,18^. In contrast to magmatic crust^19,20^, the uncoherent remanent magnetization of serpentinite limits its ability to record geomagnetic reversals, therefore resulting in weak and subdued magnetic anomalies. In contrast, seafloor generated by strong magmatic activity reveals prominent magnetic anomalies. Three states of magmatism are defined by comparison of the two conjugate ridge flanks - robust if magmatic seafloor is observed on the two flanks, intermediate if on one flank only, and starved if not observed on any flank. The alternation of these three different states along the magnetic profiles reflects the spatiotemporal variation of magmatism at the Gakkel Ridge over the last 35 Ma.

**Fig. 1:**
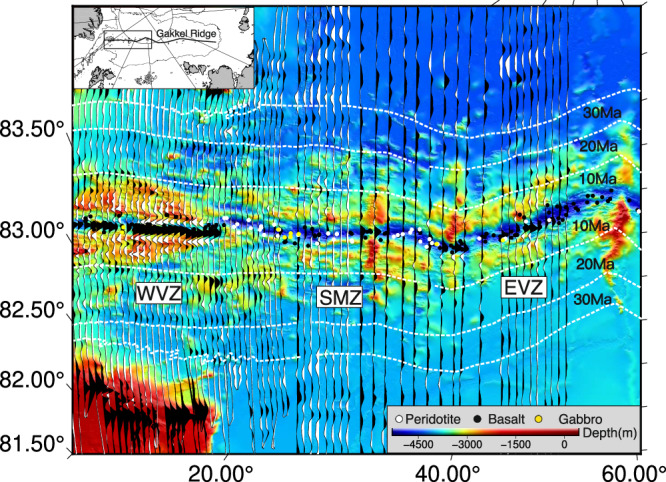
Magnetic anomalies over the bathymetry of the western Gakkel Ridge. Dashed white lines show the age at 10, 20, and 30 Ma from ref. ^55^. Black and white wiggles superimposed on the bathymetry show the positive and negative magnetic anomalies, respectively. The central magnetic anomaly is strong, disappears, and weak in the West Volcanic Zone (WVZ), Sparsely Magmatic Zone (SMZ), and East Volcanic Zone (EVZ), respectively. The white, black, and yellow dots mark locations of peridotite, basalt, and gabbro samples, respectively. The areas where peridotite and basalt were sampled correspond to subdued and strong magnetic anomalies, respectively. The insert panel shows the location of the study area.

The Gakkel Ridge (Fig. 1) spans ~1800 km in the Arctic Ocean, connecting the Lena Trough in the west off Svalbard and the Laptev Sea in the east off Siberia^12^. It has the slowest spreading rate on the Earth and is the only mid-ocean ridge not showing any prominent transform fault offsetting the spreading axis. Its full spreading rate decreases from ~13 km/Myr in the west to ~6 km/Myr in the east^21,22^. Along-axis segmentation of the Gakkel Ridge has been inferred from geophysical and geological observations. The Western Volcanic Zone (WVZ), Sparsely Magmatic Zone (SMZ), and Eastern Volcanic Zone (EVZ) (Fig. 1) correspond to different lithospheric thickness and dredged rock samples^12,14^. Isotopic geochemical analyses on basalt and peridotite samples dredged along the ridge axis indicate the coexistence of fertile and refractory mantle heterogeneities^23–26^. Compared with the Gakkel Ridge, the nature of the crust and lithosphere in most of the Eurasian Basin remains poorly known. Thick sediments in the Amundsen and Nansen basins and the ice covering the Arctic Ocean make difficult systematic geophysical and geological surveys beyond the ridge axis.

In this work, we take advantage of the variability of marine magnetic anomalies over Gakkel Ridge and adjoining basins to investigate the nature of the lithosphere beneath the thick sediments and the corresponding magmatism variation over the past 35 Ma. The results show that the strong amagmatic lithosphere occupies about two-thirds of the basins, restricts continuous magma supply and favoring episodic melt bursts.

## Results

We analyzed 60 aeromagnetic profiles over the Gakkel Ridge from 3°W to 50°E, covering the WVZ, SMZ, and EVZ (See Methods). Along the ridge axis, a prominent central magnetic anomaly appears on all profiles in the WVZ, while it is subdued or missing in the SMZ, and occurs sporadically in the EVZ. The amplitude variation of this axial magnetic anomaly is consistent with the crustal thickness variation and dredged rock samples along the ridge axis^12,14^. A thick basaltic crust, ~6 km from ref. ^27^, characterizes the volcanically active WVZ and generates the strong observed magnetic anomaly, whereas the lack of or rather thin crust (2–4 km) and serpentinite outcrops in the magma-starved SMZ do not result in any strong magnetic signal. Significant across-axis variations of the magnetic anomaly amplitude are observed as well. For example, clear magnetic anomalies occur within ~10 Ma and disappear over older regions in the WVZ. Conversely, the SMZ shows subdued and discontinuous magnetic anomalies within the recent ~20 Ma, but displays strong anomalies over older seafloor (Fig. 1). Seismic and gravity models along two short profiles across the SMZ suggest the alternation of normal and serpentinized crust and correlate well with the amplitude variation of magnetic anomalies^28,29^.

### Influence of sediment thickness

To quantitatively evaluate the spatiotemporal variation of magmatism, we conducted forward modeling on each profile applying the method of ref. ^17,18^. However, the thick sediment blanketed on the seabed hides the basement topography, which inherently affects the recorded magnetic field. To evaluate the impact of the sediment thickness that may reach up to 1000 m^30^, we built a forward model by using different top boundaries of the magnetic layer: one is the basement topography underneath the varying sediment thickness obtained from seismic reflection profiles^31^, another is the seafloor topography surveyed by the shipboard echosounder along the same seismic profile, and the last is the seafloor topography extracted from the Global Multi-Resolution Topography^32^. The location of these profiles is shown in Supplementary Information Fig. S4. The difference between these three top boundaries is bigger when the sediment is thicker, allowing to test the influence of varying sediment thickness on the observed magnetic anomaly. The dominant effect on the modeled anomalies is the contrast between the strongly magnetic magmatic seafloor and the non-magnetic amagmatic one. Differences in the geometry of the top boundary of the magnetic layer only result in negligible variations of the modeled anomaly, which is due to the large distance between the magnetic source and the aeromagnetic observation plane (>4 km bathymetry, 300 m flying altitude, and <1 km sedimentary thickness), in turn smoothing the short-wavelength magnetic signal (Supplementary Information Section 4). This test illustrates that the sediment thickness has only a minor influence on the recorded aeromagnetic data, and suggests that magnetic models based on the seafloor topography allow to constrain the type of crust beneath the sediment.

### Modeling results of three representative profiles

**Fig. 2:**
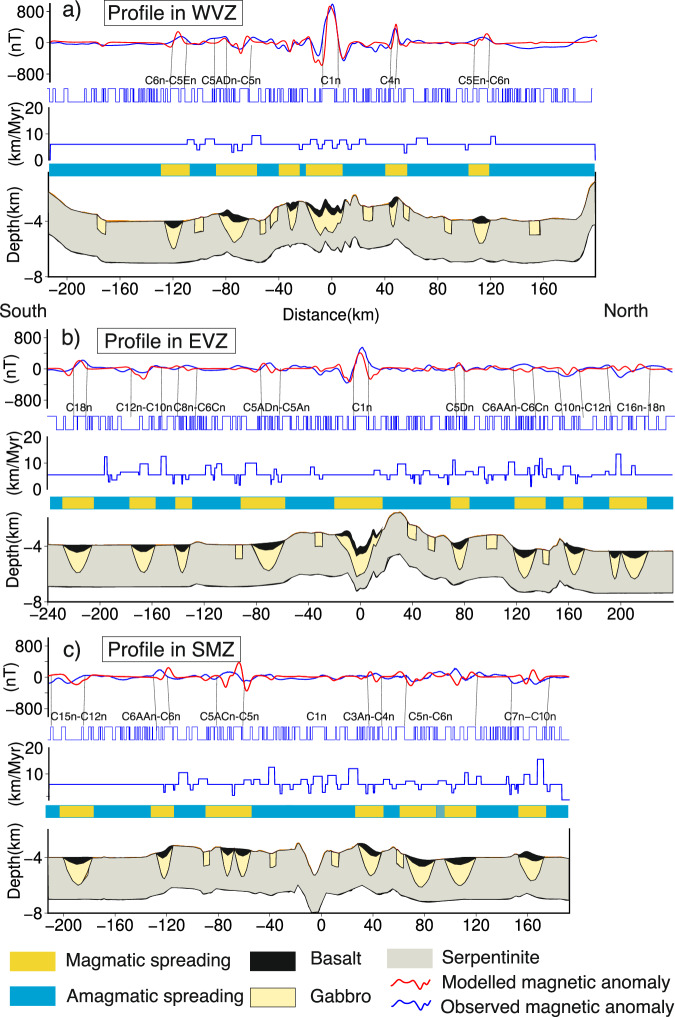
Modeling results of three representative profiles. **a** West Volcanic Zone (WVZ), **b** East Volcanic Zone (EVZ), **c** Sparsely Magmatic Zone (SMZ). In each figure, the top panel shows the observed (blue) and synthetic (red) magnetic anomalies; the two panels in the middle show the magnetic polarity reversals obtained from ref. ^50^ and a half spreading rate variation; the two panels at the bottom are the types of seafloor and the crustal section, as indicated in the legend. Black, light yellow, and gray patterns represent basalt, gabbro, and serpentinite, respectively. Dark yellow and blue patterns represent magmatic and amagmatic spreading modes, respectively. For profiles from top to bottom, the Root Mean Square (RMS) errors between modeled and observed anomalies are 105, 82, 107 nT, respectively; The assumed mean spreading rates are 12, 12, and 11 km/Myr, respectively; The mean half spreading rates (and variance of the spreading-rate perturbations) in the models are 6.2 (1.48), 5.3 (6.7), 6.5(5.1), km/Myr, respectively.

We selected three profiles as case studies that show the varying states of magmatism in time and along the ridge axis over the three major segments of the Gakkel Ridge. Figure 2a exhibits a profile over the WVZ, where the magmatic seafloor in the axial region within ~4 Ma records a strong central magnetic anomaly. In the off-axis area, the magnetic anomaly shows strong amplitude variations. Although hydrothermal alteration is reducing the crustal magnetization^33^, well-defined anomalies are still visible with lower amplitude as long as the magmatic crust exists. For example, on the northern flank (right side), magnetic anomalies show small variations between C3n and C4n with an amplitude lower than 50 nT, but become stronger again during C4n with an amplitude reaching 500 nT. The large amplitude variation of these magnetic anomaly is inconsistent with the observed topography and frequent geomagnetic magnetic reversals if a uniform magnetized source is considered (Supplementary Information Section 2). Instead, an amagmatic seafloor with gabbro intrusions or small volcanic patches during the C3n-C4n interval and a short magmatic seafloor spreading event during the C4n explain these observations well. Gabbro intrusions or small volcanic patches bearing moderate magnetization (see Methods) within the amagmatic lithosphere are used to model small magnetic anomalies that are too narrow and weak to be due to magmatic seafloor. Further away from the ridge axis in even older lithosphere, a long episode of amagmatic seafloor spreading spanning 16 million years (C4r to C6Cn) is only interrupted by a few small volcanic patches and a magmatic spreading interval during C6n. Observed magnetic anomalies not only reflect the geomagnetic polarity interval they are located on, but also integrate the effect of adjacent polarity intervals as well into broader, less resolved anomalies. This consequence of convolving the geomagnetic polarity time scale with a punctual magnetic source model^34^ is an inevitable difficulty for magnetic anomaly modeling at the ultraslow spreading ridge, for which a resolution better than 1 Myr cannot be reached. Frequent magnetic reversals such as the C5n-C5Cn and C6AA-C6Cn intervals may result in subdued magnetic anomalies at an ultraslow spreading rate, possibly leading to overestimating the abundance of amagmatic seafloor for these time intervals. However, we observe notable variations on the conjugate southern flank, suggesting that this effect is negligible. Magnetic anomalies recorded over magmatic crust in time intervals with frequent reversals may integrate several reversals within a single anomaly at a slow or ultraslow spreading rate. However, the distribution of amagmatic seafloor within 2 Myr-long intervals along the investigated magnetic profiles shows no statistical bias toward time intervals with frequent magnetic reversals (Supplementary Information Fig. S3), confirming that this effect is negligible.

A profile in the EVZ exhibits similar features (Fig. 2b). The central magnetic anomaly spreads over ~3 Ma but displays an amplitude ~200 nT lower than that of the same anomaly on the profile in the WVZ discussed above. Off-axis, the magmatic seafloor appears sparsely for time intervals C5An-C5Cn, C6Cn-C8n, C10n-C12n, and C18n-C19n on the southern flank and C5Cn, C6AAn-C6Cn, C10n-C12n, and C16n-C19n on the northern flank. Before C6Cn (~24 Ma), magmatic crust was more abundant and generated stronger magnetic anomalies, while sparser and weaker magnetic anomalies are observed from C6Cn to the present. The alternation of magmatic and amagmatic seafloor spreading is faster, and the duration of magmatically robust episodes is shorter for this period. A possible reason is that the slight ridge obliquity and slower full spreading rate in this eastern area restricts the effective magma supply, shortening the magmatic episodes^35^. The faster magmatic variations in this segment induce a more complex crustal structure and make it difficult to achieve a perfect fit of our model for each anomaly, especially off-axis, where information on the age, spreading rate, and local tectonic history is limited and geological parameters such as the degree of basalt alteration and crustal faulting are poorly constrained. However, even imperfect, our modeling aims at identifying regionally the types of seafloor by primarily relying on the amplitude of prominent magnetic anomalies.

Conversely, the profile over the SMZ (Fig. 2c) shows an amagmatic seafloor in the axial region up to ~7 Ma. This magma-starved episode is quite recent with respect to the 35 Myr of spreading history and was preceded by magmatic seafloor, as observed off-axis. Long weakly magmatic or amagmatic episodes increase the difficulty to fit models to the magnetic anomalies. Possible existence of detachment faults may induce asymmetric spreading in short time windows (<2 Myr), but in the long term, the seafloor age does not depart much from a general symmetry on the two flanks of the ridge^2^. Additionally, the zero-age point of the model is hard to set without reference to the central magnetic anomaly, resulting in the synthetic anomaly being slightly shifted laterally, although having negligible effects on identifying the types of seafloor regionally. Modeling the three profiles described above shows that magmatic seafloor does not correlate well with the basement topography^31^, as shallow topography may either correspond to volcanic seafloor or Oceanic Core Complexes (OCCs).

### Types of seafloor and magmatism variation

**Fig. 3:**
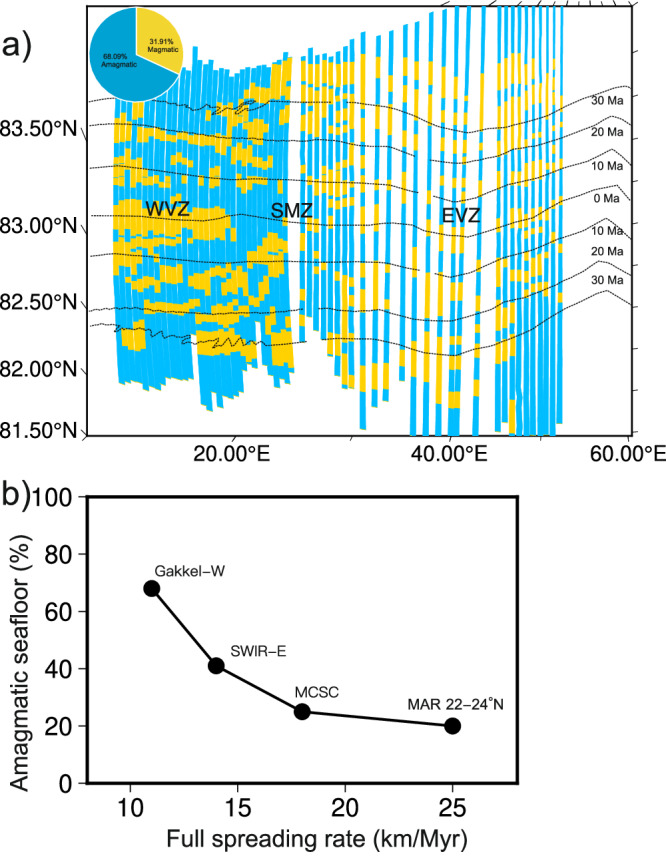
Distribution of different types of seafloor. **a** A Seafloor classification along the magnetic profiles in the study area, in which blue represents an amagmatic seafloor contrasting with magmatic seafloor in yellow. Amagmatic seafloor accounts for 68% of the total seafloor, in contrast to 32% for magmatic seafloor. The dashed black lines show the age obtained from the magnetic models of ref. ^55^. WVZ West Volcanic Zone, SMZ Sparsely Magmatic Zone, EVZ East Volcanic Zone. **b** Ratios of amagmatic seafloor for ridges versus spreading rate for different spreading centers. MAR22-24°N: Mid-Atlantic Ridge 22°−24°N, 22 km/Myr^36^; MCSC Mid-Cayman Spreading Center, 18 km/Myr^15^; SWIR-E Eastern section (61-66°E) of the Southwest Indian Ridge, 14 km/Myr^8^; Gakkel-W Western section (3°W-60°E) of the Gakkel Ridge, 12 km/Myr.

We located the different types of seafloor derived from the modeling of each profile in map view to unravel their geographical distribution. For regional characterization, we interpolated the points sampled along profiles onto a grid and used values of 1 and 0 to indicate magmatic and amagmatic seafloor, respectively, whereas the gaps between the profiles were filled with a NaN value. The ratio of magmatic and amagmatic seafloor were then calculated, showing that amagmatic seafloor accounts for 68% of the total seafloor in contrast to 32% for the magmatic seafloor, characterizing the seafloor spreading history in the Arctic Ocean for the last ~35 Ma (Fig. 3). Detachment faults cover 20–25% of the total seafloor surface of the Central Mid-Atlantic Ridge^36^, 25% at the Mid-Cayman Spreading Center^15^, at least 41% at the Easternmost Southwest Indian Ridge^8^, and up to 70% at the Australian-Antarctic Discordance, an anomalously cold section of the intermediate spreading Southeast Indian Ridge^37^. Although magmatism is highly variable along segments of the ultraslow spreading ridges, the amagmatic seafloor becomes more and more prevalent when the spreading rate decreases. According to the thermal structure predicted from the passive upwelling model for different spreading rate^38^, the slowest-spreading Gakkel Ridge should be much colder than the other ridges, hence explaining the 68% of amagmatic seafloor. However, some short volcanic intervals, less than 1Myr-long or within a period of frequent magnetic reversals, may not be taken into account.

The full spreading rate of the Gakkel Ridge decreases between 13 km/Myr and 6 km/Myr^22^ from west to east^21^. The modeled profiles also encompass this spreading rate variation, ranging from ~14 km/Myr west of WVZ to ~10 km/Myr east of the EVZ, in general agreement with the predicted spreading rate range of 13–11 km/Myr derived from plate tectonic models^22^. Our aeromagnetic profiles are parallel to the spreading direction predicted by these models^22^ for the Gakkel Ridge. At first order, the finite rotation describing the relative motion of North America and Eurasia did not vary much over the last 20^39^ and 45^40^ Ma, allowing us to consider that the aeromagnetic profiles follow flowlines of the relative plate motion and do therefore not require any projection. We observe a slight asymmetric spreading in the EVZ, where the southern flank spreads 0.5 km/Myr faster than the northern flank.

**Fig. 4:**
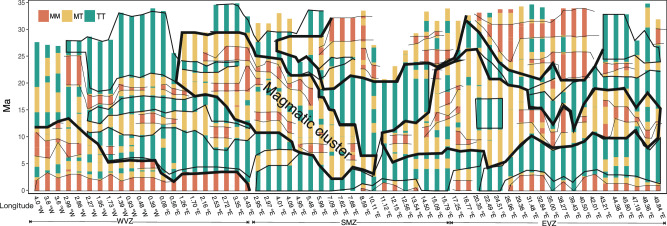
Temporal variation of magmatism along each across-axis magnetic profile. Red represents robust magmatism, with magmatic seafloor on both flanks (MM); Yellow intermediate magmatism with one magmatic flank and the conjugate amagmatic flank (MT); Green starved magmatism with amagmatic seafloor on both flanks (TT). Thin lines mark melt bursts, thick lines delineate possible clusters related to a more abundant fertile mantle.

Knowing the different types of seafloor versus age for each profile, we derived the state of magmatism (robust, intermediate, and starved, marked in red, yellow, and green, respectively, in Fig. 4) by considering the type (magmatic or amagmatic) of coeval seafloor on conjugate flanks (see above; ref. ^18^). The width of the amagmatic domains varies from ~20–150 km and their longevity ranges from ~3–10 Myr. Compared with the prevalent amagmatic seafloor, the robust magmatic domains show shorter longevity, ranging from ~1–5 Myr, but display a comparable width of ~20–200 km. Lateral migration of the melt, although limited within the cold lithosphere of an ultraslow spreading ridge^2^, may enlarge the area affected by magmatism. Short intervals of robust magmatism are usually followed by a longer, up to 10 Myr-long interval of intermediate magmatism, before returning to the starved magmatic state.

## Discussion

### Melt Bursts within widespread amagmatic lithosphere

**Fig. 5:**
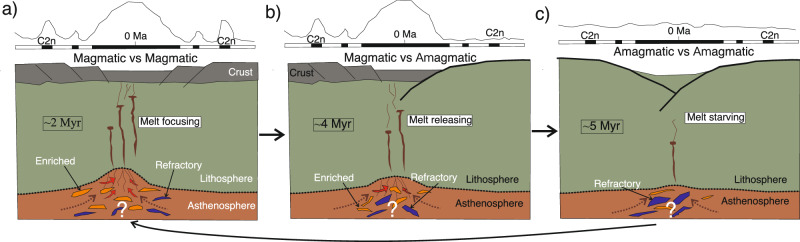
Magmatic sequence. **a** Melt focusing at the base of the lithosphere results in robust magmatism with magmatic seafloor and strong magnetic anomalies on both flanks. **b** Reduced melt release results in intermediate magmatism sustaining magmatic spreading on one flank only; magnetic chrons are not observed on the flank with amagmatic spreading. **c** All melt has been released, resulting in starved magmatism and amagmatic seafloor on both flanks with no magnetic anomaly observed. In each panel, from top to bottom, the white (reversed) and black (normal) columns show the geomagnetic polarities; Dark gray, green, and red patterns represent crust, lithosphere, and asthenosphere, respectively. In the asthenosphere, orange and blue patterns represent enriched and refractory mantle components, respectively. When the melt segregated from the fertile mantle is sufficient, robust magmatism resumes, and the sequence starts again. The mantle composition may modulate the sequence, with more melt generated when the fertile mantle component is more abundant in the melting zone.

The above investigation shows that an amagmatic lithosphere is widespread in the Amundsen and Nansen basins adjoining the Gakkel Ridge. Conversely, magmatic seafloor is sparse within these basins, with robust magmatic bursts only happening episodically. The mechanism controlling such prominent temporal variations of the magmatic activity in this confined basin, without any known hotspot or “cold spot” such as large transform faults during the last 45 Ma, is still poorly understood. Seismic studies along the Gakkel Ridge discovered a very thick lithosphere, possibly up to 35 km, for the amagmatic SMZ^14^. The strong lithosphere acts as a barrier and restricts the melt transport, resulting in the melt ponding at the base of the lithosphere until it is in sufficient amount to burst^41^. Such a mechanism, although not at the same scale, may explain bursts of magma supply at the eastern SWIR and a volcano in the eastern domain of the Gakkel Ridge at 85°E^42^. The temporal variations of magmatism shown in Fig. 4 suggest that a short phase of robust melt emplacement, roughly spanning 2 Myr, is always followed by a phase of intermediate magmatism, ~4 Myr-long, and a phase of starved magmatism ~5 Myr-long (Fig. 5). This sequence suggests the following melt supply dynamics in the lithosphere: (1) The focused melt accumulates at the base of the lithosphere until the burst, initially sustaining a pure magmatic seafloor spreading for ~2 Myr; (2) With the melt release and cooling of the new lithosphere, the amount of melt decreases, sustaining magmatic accretion on one flank and mantle exhumation on the conjugate one for ~4 Myr; (3) After all the accumulated melt is released, widespread amagmatic seafloor spreading thoroughly takes over. During this sequence, some melt might be trapped in the cold lithosphere, forming small patches of magmatism within the amagmatic seafloor domains.

### Active mantle upwelling results in the focused melt

Similar melt burst events are also observed at the eastern Gakkel Ridge near 85°E. Crustal thickness is ~8 km at segment centers, where volcanoes are hosted, and a mechanism of active mantle upwelling is proposed to support the episodic melt bursts that cannot be achieved solely by a passive mantle upwelling model^16^. Focusing of melt might be driven by active mantle upwelling reflecting changes in the mantle temperature or composition in the melting zone, increasing the crustal thickness and magnetic amplitude. Whether any similar active mantle upwelling existed beneath the past magmatic bursts shown by our magnetic study cannot be proven because the mantle dynamics keep changing, and the current state of the melting zone beneath the ridge axis cannot represent the past. However, the similarities between these past magmatic bursts and the present one observed on the same spreading center at 3°W and 85°E suggest that the same processes were at work and active mantle upwelling was probably happening episodically in the whole area for the last 35 Ma, over time intervals of roughly 10 Myr. Meanwhile, the melt bursts depicted by the magnetic profiles are not happening randomly and independently but seem to be spatially and temporally related and form clusters, for example starting on profiles 27–33 at 28 Ma, enlarging to profiles 27–39 at ~20 Ma, then receding to profiles 37–43 at ~10 Ma, and ending at profiles 41–42 at ~2 Ma (Fig. 4). These clusters may reflect compositional or physical variations in the mantle.

### Contribution of mantle heterogeneity?

Geochemical isotopic composition of the rock samples, such as the Re-Os of ultramafic peridotite and Hf-Nd of basalt, indicates the existence of an ancient recycled lithospheric mantle component within a heterogeneous mantle system beneath the axis of the Gakkel Ridge^23–25,43^. This ancient residual mantle does not participate in the current melt generation but may contribute to the widespread amagmatic lithosphere. A recent study combining isotopic variations and shear-wave velocity structure of the Mid-Atlantic Ridge evaluated that the recycled refractory mantle represents ~67% of the upper mantle^44^. Our study reveals that the amagmatic seafloor makes ~68% of the total seafloor at the Gakkel Ridge (Fig. 3), where the refractory mantle may play a significant role in reducing the magma supply. In this case, the “marble-cake” mantle^45^ made of refractory and fertile components generates a variable amount of melt depending on the proportion of each component involved in the melting zone. When the fertile mantle is abundant, more melt is segregated and focused at the base of the lithosphere. After the segregated melt is released, the refractory mantle is widespread, then decreasing the melt generation and reducing the active mantle upwelling, until the fertile mantle component becomes abundant again. Although such a contribution of compositional mantle heterogeneities to the melt supply probably exists at most spreading centers, the ultraslow spreading context makes it visible through shallow crustal observations^46^. The more frequent bursts observed in the magmatic clusters suggest that they correspond to regions with more abundant fertile mantle (Fig. 5).

Variations of mantle temperature may be another factor affecting the melt generation^16^, which induces temporal variations of magmatism as well. However, without any hot spot or “cold spot” able to modify the mantle temperature and in the context of a confined basin, it is not easy to explain hundreds-kilometer-scale episodic mantle temperature variations with mantle convection alone. Additionally, recent geochemical studies in the eastern sections of this ridge favor mantle fertility as the key factor controlling the crustal thickness, instead of variation of mantle temperature^47^.

Our study expands current understanding of the nature of the lithosphere formed at the Gakkel Ridge until 35 Ma, overcoming the difficulties of systematic rock sampling in an environment where the basement is blanketed by thick sediments. The striking dominance of amagmatic seafloor spreading indicates a strong lithosphere along the axis of this ultraslow spreading ridge. Sequences made of a short phase of robust magmatism, followed by longer phases of weaker magmatism, then starved magmatism, reveal the dynamics of melt segregation and transport beneath and within the lithosphere. This episodic magmatism, consistent over the whole studied section of the Gakkel Ridge, suggests that the mantle heterogeneity may exert a control on the variation of magmatism as depicted by the strong variability of marine and airborne magnetic anomalies over the slow and ultraslow spreading ridges.

## Methods

### Aeromagnetic data processing

The aeromagnetic data analyzed in this paper were collected by Project Magnet in 1974-1975^21^. The airborne data were acquired 300 m above the sea surface with a 250 m sampling interval and 9–17 km line spacing. Navigation, cross-correlation leveling, filtering, and IGRF (International Geomagnetic Reference Field^48^) corrections were previously applied (Supplementary Information) to overcome any navigation problem and influence of external fields. We only removed long-wavelength trends from the archived data. The corrected magnetic anomaly correlates well with a dataset collected in 1998–1999 with GPS navigation (Supplementary Information Fig. S1). Both the location and amplitude of the magnetic anomaly at crossing points of the two independent datasets are consistent.

### Modeling the types of seafloor from the variation of magnetic anomalies

We modeled the magmatic and amagmatic seafloor for 60 profiles based on the variation of magnetic anomalies by using the method of ref. ^17,18^. At ultraslow spreading centers, magmatic and amagmatic crust present contrasted structure and magnetic anomalies. In the model, magmatic crust made of basalt, gabbro and serpentinite layers is assigned to recover the strong magnetic anomaly, whereas amagmatic crust with gabbro intrusions or small volcanic patches embedded in a pure serpentinite layer corresponds to subdued magnetic anomalies. Basalt and gabbro bear remanent magnetization of 20 and 2 A/m, respectively. The demagnetization of basalt resulting from the alteration process is modeled by a linear decay from the ridge axis to the profile end. When gabbro is outcropping, such as in the detachment-fault footwall, its magnetization is 3 A/m, slightly stronger because the faster cooling of exhumed gabbro bodies induces a higher fractionation rate and iron concentration^49^. The serpentinite layer bears a low induced magnetization of 0.5 A/m, which does not record magnetic reversals^19,20^.

The maximum thickness *T*_*m*_ of basalt, gabbro, and serpentinite layers during a magmatic spreading episode are 500 m, 1500 m, and 1000 m, respectively. The thicknesses of the basalt and gabbro layers decrease from the center of the magmatic segment towards its boundary with the amagmatic crust, following the function:

1 $$T={T}_{m}\times \cos \left(\left|\frac{x-\frac{a+b}{2}}{\frac{a-b+w}{2}}\right|\times \frac{{{{\rm{\pi }}}}}{2}\right)$$

Where *T* is the thickness at distance *x* from the ridge axis, *a* and *b* are the start and end points of the magmatic crust, and *w* is a weight parameter that modulates the width of the transition zone between magmatic and amagmatic crust. During an amagmatic spreading episode, a 3-km serpentinite layer and occasional 1-km-thick gabbro intrusions are considered.

The geomagnetic polarity determining the remanent magnetization vector of the magmatic crust is set up referring to ref. ^50^. The age of the seafloor is initially calculated by the distance from the ridge axis and a given half-spreading rate. When the half-spreading rate varies in some time intervals, the crustal age will also slightly change.

With these parameters, we can calculate the synthetic magnetic anomalies along magnetic profiles. The subseafloor space is divided into prism units with uniform scale and magnetization. The total field magnetic anomaly is then calculated by:

2 $$\bigtriangleup T={\sum}_{i=1}^{N}{M}_{i}{K}_{i}(x,y,z,I,D,{I}_{0},{D}_{0})$$

Where *K*_*i*_ is the analytical kernel function for a uniformly magnetized rectangular prism following ref. ^51^. *M*_*i*_ is magnetization intensity of each prism unit; *x*, *y*, *z* describe the prism location; *I* and *D* are the inclination and declination of the magnetization vector; and *I*_*0*_ and *D*_*0*_ are those of the ambient magnetic field. In our polar area, *I* = *I*_*0*_ = 90°, *D* = *D*_*0*_ = 0°.

During the modeling procedure, the spatial distribution of magmatic crust and the spacing of magnetic chrons are iteratively adjusted until the synthetic anomaly closely matches the observed data. From these iterations, the half-spreading rate of each ridge flank is determined over time, allowing for the reconstruction of spreading asymmetry as a time-dependent variable. The averaged half spreading rate varies 6.5 km/Myr in the west to 5 km/Myr in the east. Specifically, asymmetric spreading is also observed in the east, where the southern flank shows a faster half-spreading rate of 5.5 km/Myr compared with the 5.0 km/Myr for the northern flank.

## Supplementary information

Supplementary Information

Transparent Peer Review file

## Source data

source data

## Data Availability

Raw aeromagnetic data, collected by the Project Magnet, used in this paper, can be accessed at the NCEI database. The bathymetry data can be accessed by ref. ^52^(10.1038/s41597-024-04278-w). The magnetic profile data generated in this study have been deposited in the general repository Zenodo database via ref. ^53^(10.5281/zenodo.20758198). Source data are provided with this paper.

## Code availability

The code of magnetic models and related data for each profile can be accessed from the Zenodo repository database via ref. ^54^ (10.5281/zenodo.20794014).
