# Supplementary material for: Melt burst events in a dominantly amagmatic lithosphere at Gakkel Ridge in the Arctic Ocean
Source: Nat Commun. 2026 Aug 7;17:8009. doi: 10.1038/s41467-026-76409-3 (PMC13451187; doi:10.1038/s41467-026-76409-3)
Supplement: Supplementary file 1 [file 41467_2026_76409_MOESM1_ESM.pdf]

Supplementary Information for:

Melt burst events in a dominantly amagmatic lithosphere at Gakkel Ridge in the Arctic Ocean

Fei Zhou[1], Ingo Grevemeyer[1], Jérôme Dyment[2]

1. GEOMAR Helmholtz Centre for Ocean Research Kiel, Kiel, 24148, Germany

2. Université Paris Cité, Institut de physique du globe de Paris, CNRS, 75238 Paris, France

Contents of the file:

Section 1: Validation of the data quality

Section 2: Comparison of models with uniform and patchy magnetic layers

Section 3: Statistic result to show that the frequent magnetic reversal period does not generate overestimate for the amagmatic seafloor in the magnetic models.

Section 4: Test on influences of the magnetic layers with different top boundaries and different crustal structures on the marine magnetic anomalies.

Section 1.

The aeromagnetic data used in our study were collected in 1974-1975 when the GPS navigation was not applied yet. Therefore, the original data may suffer from the navigation problem that should be corrected before conducting any further analysis about crustal magmatism. We acquired the dataset in the NCEI database. The dataset used in our study was obtained from the NCEI (ex-NGDC) GEODAS database. It has been processed and corrected by researchers from the Naval Research Laboratory, Skip Kovacs at the Atlantic Geoscience Centre (AGC) and Volodja Glebovsky in Dartmouth, Nova Scotia in November 1993 before the data were included in the database. The corrections include shifting, cross-correlation leveling, filtering, and IGRF corrections (NCEI database https://www.ncei.noaa.gov/maps/trackline-geophysics/). Navigation of the old data was handled and calibrated using the Thule_1992 dataset collected with differential GPS navigation. Any potential bias was therefore considered and corrected by leveling and filtering before the data were included in the database in November 1993. After extracting the data from the NCEI database, we only removed a linear trend of the data to obtain clearer contrast. We compared the data used in our study with a more recent dataset acquired by the US Navy in 1998 and1999. The crossing points of the two datasets correlates well both in the axial and off-axial areas (Red boxes in Figure S1). The central magnetic anomaly is over the ridge axis -strong over the West Volcanic Zone, missing in the Sparsely Volcanic Zone, and occasionally seen in the Eastern Volcanic Zone- which is consistent with the sampled rock types along the ridge axis. Off-axis, strong magnetic anomaly are depicted over the Yermak Plateau and the Morris Jesup Rise. The navigation and amplitude variation of the dataset both along and across the ridge axis appear to be sufficient to investigate the variations of magmatism.

To be more quantitative, we calculated the cross-over errors (COE) between the two data sets. Among 1718 cross-over points, the mean difference between them is 11nT, with a standard deviation of 78nT and Root Mean Square (RMS) error of 79nT, which is negligible comparing with the amplitude of observed magnetic anomalies ranging from ~-700 to1500 nT.

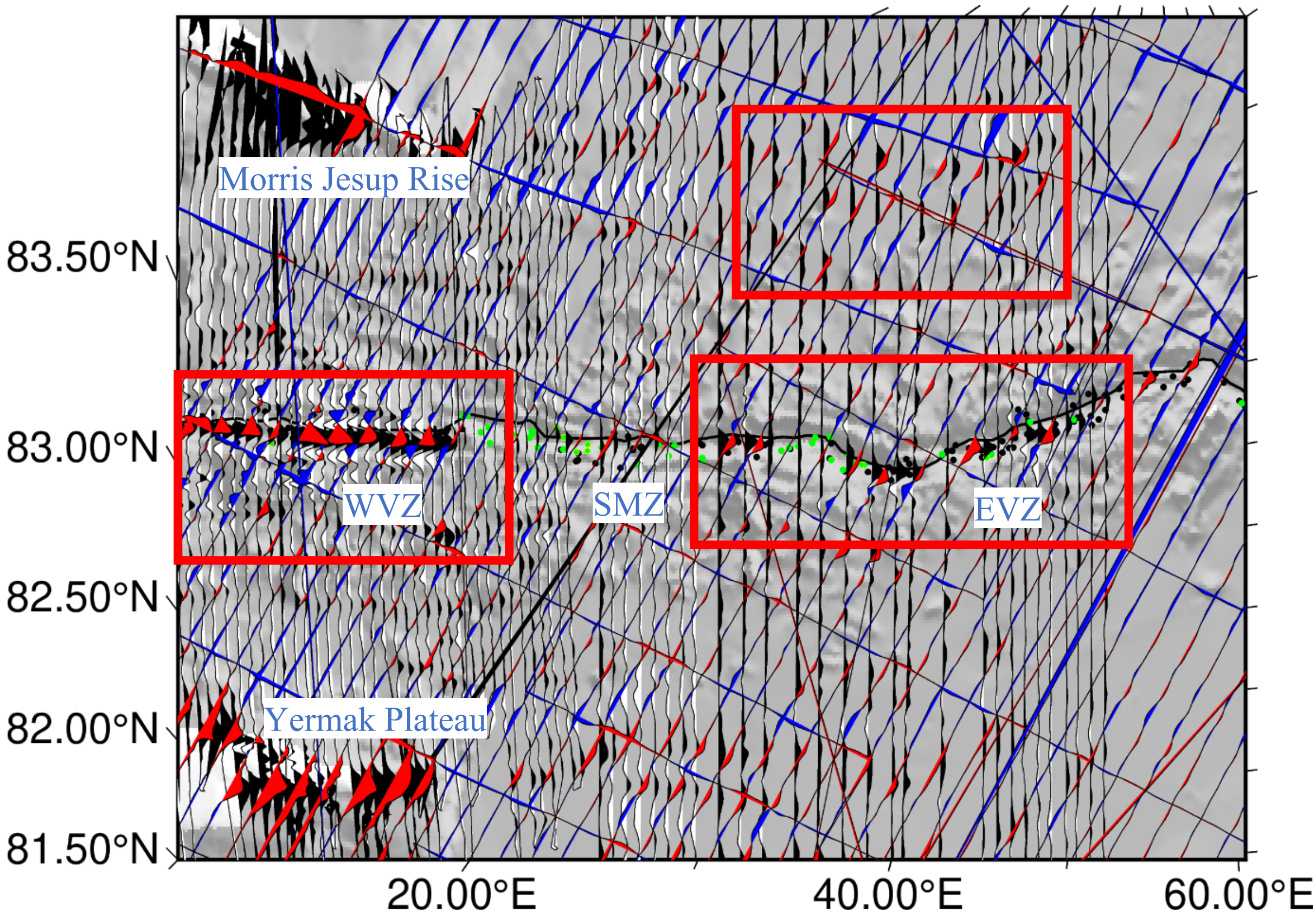

Figure S1. Comparison of the two aeromagnetic datasets collected in 1970s (black and white wiggles) and 1990s (red and blue wiggles). The solid black line marks the location of the Gakkel Ridge axis. The black and green dots show the locations of basalt and peridotite, respectively. The red boxes delineate the areas that clearly show the consistency between the two datasets.

Section 2.

In this section, we compare our models with models assuming constant crustal thickness to indicate the advantage of the "patchy" crustal model for modeling the complex marine

magnetic anomalies over ultraslow spreading ridges. We produced synthetic magnetic anomalies for the three real profiles shown in Figure 2 of the paper. In Figure S2, on the right, we assume a constant-thickness magnetic layer, and on the left we just duplicated the "patchy" approach shown in Figure 2 of the manuscript. The geomagnetic polarity timescale is the classic Cande and Kent (1995) and the spreading rate was calculated from global plate motion calculator (University of Tokyo http://ofgs.aori.u-tokyo.ac.jp/~okino/platecalc_new.html) that integrates current existing NUVEL and MORVEL models. From top to bottom, the RMS residual between observed and synthetic anomalies are 277, 234, and 178 nT, respectively, for the constant thickness layer models and 105, 107, and 82 nT, respectively, for the patchy approach, i.e., two to three times larger under the assumption of a constant thickness layer, justifying our search for more suited models to fit the observations. The approach with uniform magnetic layer may work well for the fast-spreading ridges with nearly constant crustal thickness, while the patchy model is more suitable for the ultraslow-spreading ridges where the crust is always missing.

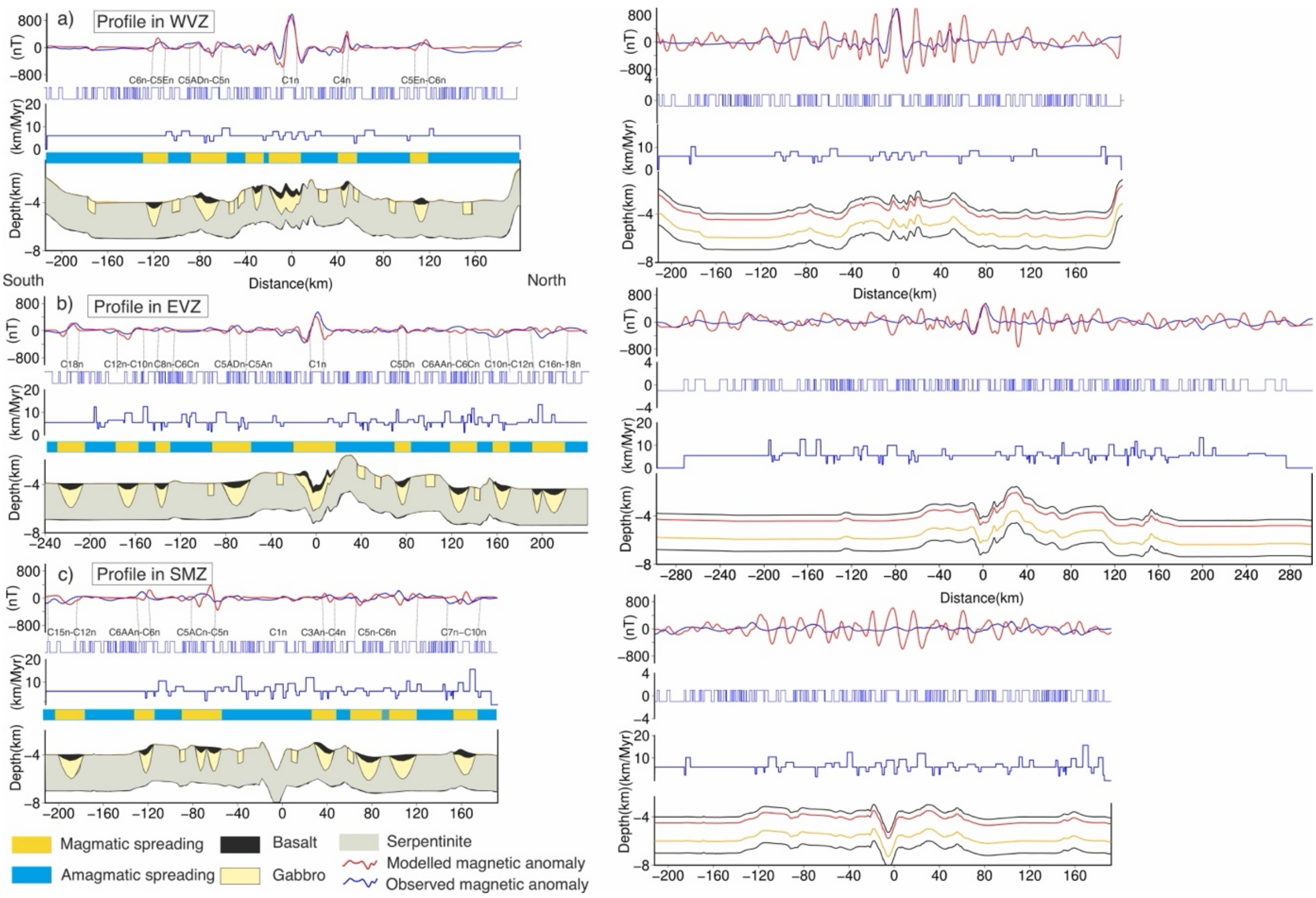

Figure S2: synthetic magnetic anomalies on representative profiles across the WVZ (top), EVZ (middle) and SMZ (bottom)sections assuming (left) our patchy approach duplicated from Figure 2 of the manuscript, and (right) a uniform thickness magnetic layer.

Section 3.

In this section, we test whether the types of seafloor obtained from the magnetic models show a preference for frequent magnetic reversal period. We count the points along magnetic profiles where amagmatic seafloor is modeled in each 2-Myr period. The result shows that the modeled amagmatic is not preferential to the frequent magnetic reversal period such as the period between C6AA-C6Cn (Figure S3), indicating a negligible overestimate for magmatic seafloor in the magnetic models.

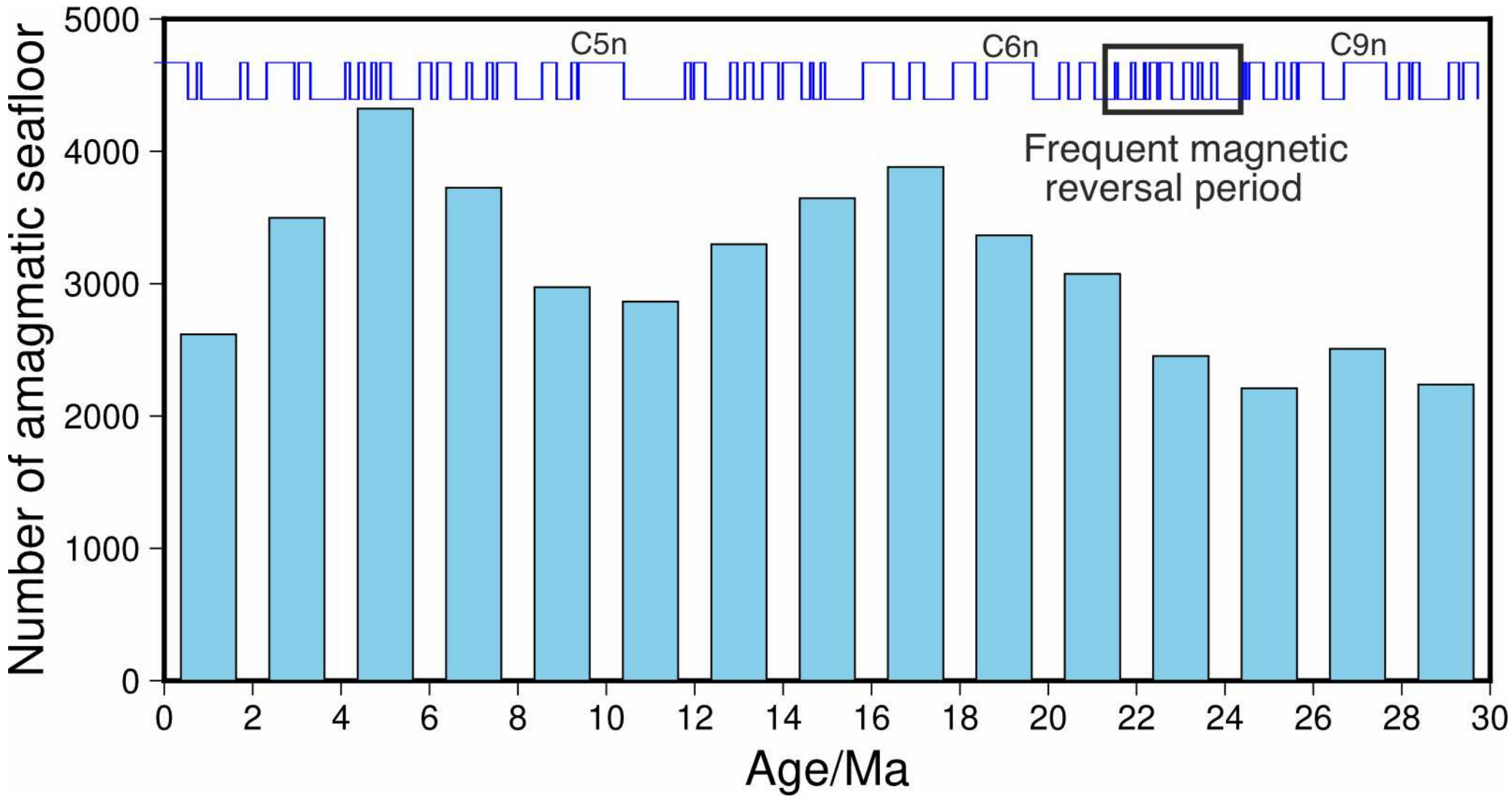

Figure S3. Statistic result counting the distribution of amagmatic seafloor in each 2-Myr interval along the investigated magnetic profiles. The top panel shows the magnetic polarity reversals.

Section 4.

In this section, we test the influence of the thick sediment on the marine magnetic anomalies in the Gakkel Ridge. We selected a magnetic profile overlapped with a previous reported seismic profile as shown in Figure S4a. First, we model the magnetic anomaly with magnetic layers topped by the different boundaries from the MGRT bathymetry, seafloor from the seismic survey, and the basement derived from the seismic reflection. Magnetic layer is composed of a 500m-thick basalt layer and an underlying 1500m-thick gabbro layer, bearing 6 A/m and 2A/m remanent magnetization, respectively. The modeling results show that the calculated magnetic anomalies do not show significant differences in the last 30 Ma, even in the areas where the applied topography has ~1000 m discrepancy with the real basement (Figure S4b). This means that the thick sediment blanketed on the seafloor does not have accountable influence on the marine magnetic anomalies. Deep water depth in the Gakkel Ridge and the 300 m observation level above the sea surface filter the shallow signature from

the local topographic variation. The spectral analysis (Figure S5) also illustrates that the three synthetic magnetic anomalies calculated by using different top boundaries of the magnetic layer only exhibit some small differences in the low spatial frequency (long-wavelength) no matter in the amplitude spectrum, phase spectrum, or the power spectral density plots. In higher frequency domain (short-wavelength), the three datasets are nearly identical, further indicating that the short-wavelength signals generated by the topography have been filtered by the long distance away from the source layer and observation datum plane.

Second, we test the influence of varied lithospheric structure on the marine magnetic anomalies in Figure S4c. We use the same parameters for the magmatic seafloor as the model above. Amagmatic seafloor is only composed of a serpentinite layer with weak induced magnetization. The results show that during the amagmatic spreading period, magnetic anomaly is rather subdued, magnetic reversals cannot be recorded. These tests demonstrating that the hidden basement because of the thick sediment on the Eurasian basin does not generate accountable influence on the magnetic anomalies compared with the change of the seafloor spreading modes.

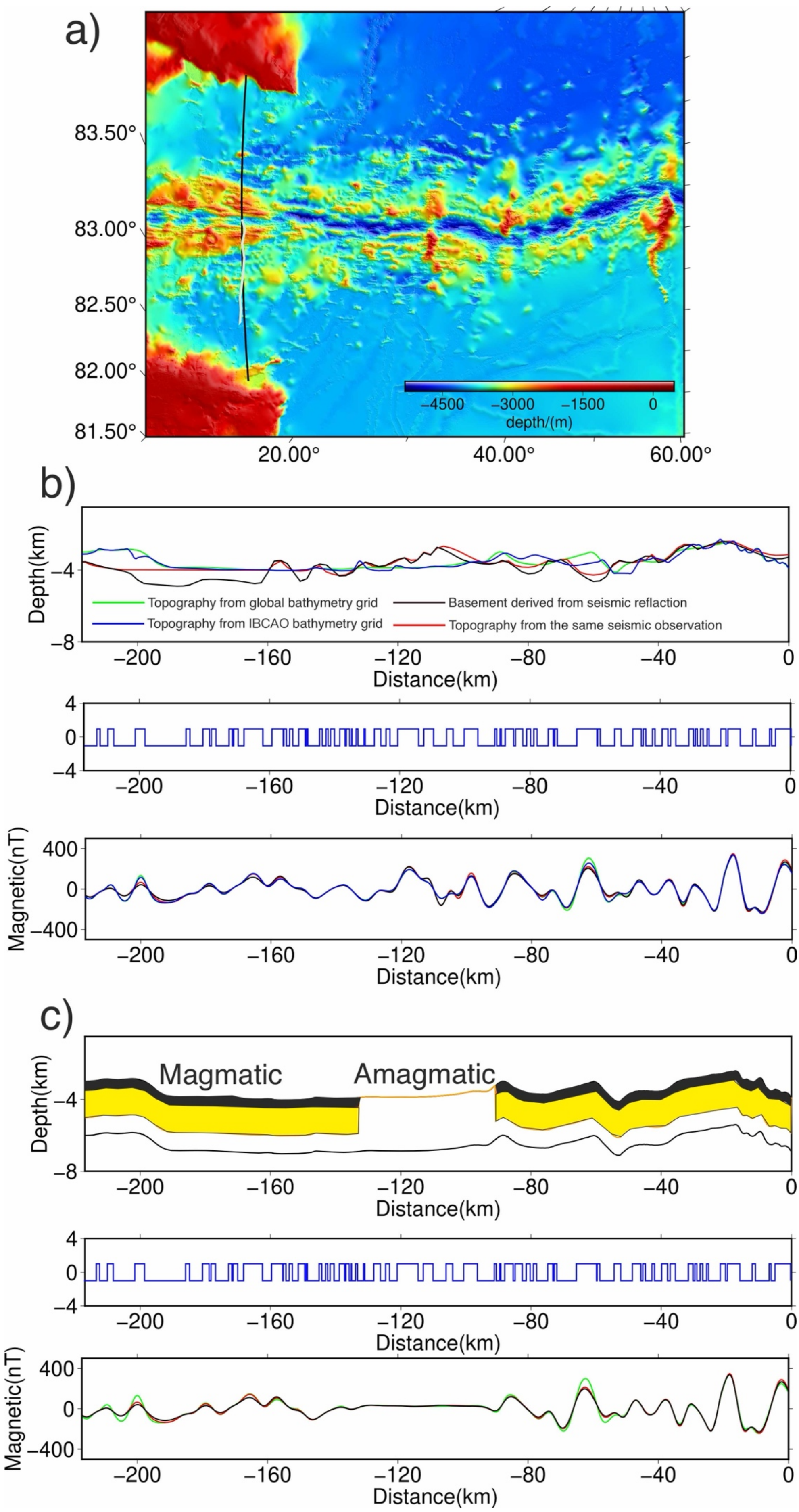
a)
83.50°
83.00°
82.50°
82.00°
81.50°
20.00°
40.00°
60.00°
−4500 −3000 −1500 0
depth/(m)
b)
Depth(km)
Topography from global bathymetry grid
Topography from IBCAO bathymetry grid
Basement derived from seismic reflection
Topography from the same seismic observation
Distance(km)
Magnetic(nT)
c)
Magmatic
Amagmatic

Figure S4. Forward models illustrating the influence of sediment and magmatic layer on magnetic anomalies. (a). Location of selected magnetic (solid black line) and seismic (solid white line) profiles. (b). Magnetic anomaly calculated from magnetic layers topped by the topography from MGRT bathymetry data (green), seafloor from IBCAO bathymetry data (blue), seafloor from seismic survey (red), and basement derived from seismic reflection (black). The medium panel shows the magnetic reversals; the bottom panel shows the magnetic anomalies. The colors are same as the corresponding topography shown in the top panel. (c). Influence of the lack of magmatic layer on the marine magnetic anomalies. Colored areas are the magmatic crust, the white area in between shows the amagmatic crust which cannot record magnetic reversals.

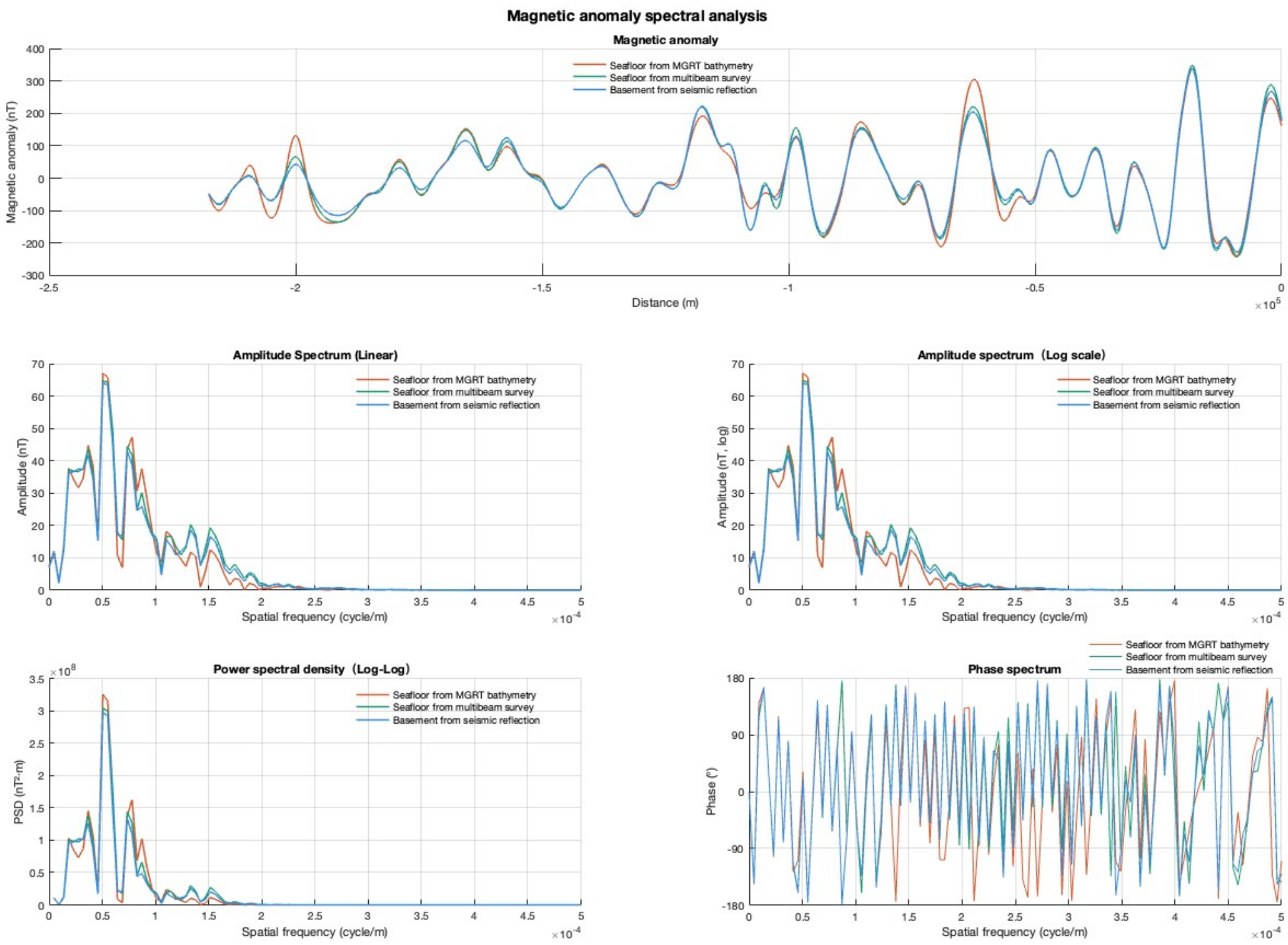

Figure S5. Spectral analysis of the magnetic anomalies calculated by using different top boundaries of the magnetic layer.
