# Supplementary material for: Melt burst events in a dominantly amagmatic lithosphere at Gakkel Ridge in the Arctic Ocean
Source: Nat Commun. 2026 Aug 7;17:8009. doi: 10.1038/s41467-026-76409-3 (PMC13451187; doi:10.1038/s41467-026-76409-3)
Supplement: Supplementary file 2 [file 41467_2026_76409_MOESM2_ESM.pdf]

nature portfolio

Peer Review File

Melt burst events in a dominantly amagmatic lithosphere at Gakkel Ridge in the Arctic Ocean

Corresponding Author: Dr Fei Zhou

This file contains all reviewer reports in order by version, followed by all author rebuttals in order by version. Parts of this Peer Review File have been redacted as indicated to remove third-party material.

Version 0:

Reviewer comments:

Reviewer #1

(Remarks to the Author)
The paper addresses the variation of the magmatic activity along the ultraslow spreading Gakkel Ridge in the Arctic Ocean. The authors used analysis of historical aeromagnetic data to correlate the intensity of the magnetic anomalies with the periods of magmatic production at the spreading axis. They performed magnetic modeling of the oceanic crust from between 35 Ma and the present time on both sides of the ridge axis. The analysis addressed the intensity and symmetry of the seafloor spreading magnetic anomalies. They concluded that sequences of the robust magmatism lasted about two million years and were followed by longer phases of weak magmatism and then by the starved magmatic regime. These cycles have been suggested to reflect the dynamics of the melt segregation and transport within the thick lithosphere of the ultraslow Gakkel Ridge. According to their estimates, 68% of the oceanic crust in the Eurasia Basin was formed in the starved magmatic accretion regime.
The magnetic modeling was performed on the airborne magnetic profiles collected in the middle of the 70s by the U.S. NRL. Without satellite navigation at that time, it was not uncommon for airborne data collected to suffer from navigation problems. Therefore, some anomalies in old surveys can be shifted or be narrower or wider than they are due to poorly controlled positioning and speed. Another problem with magnetic data in the Arctic is the strong diurnal variation of the geomagnetic field. The geomagnetic storms and ionospheric currents can produce short-period variations of the geomagnetic field undistinguishable from geological anomalies. For aeromagnetic surveys, we just hope that the aircraft has a high enough speed such that the geomagnetic variation would be recorded as a linear trend. The standard processing would include data from the magnetic reference stations and tie-line levelling. In the Arctic Ocean, the use of base stations close enough to the survey area is logistically difficult. Therefore, we rely mostly on the levelling procedures using data from the crossing points with tie lines. The residuals at the crossing points with tie lines are used to determine and remove a linear (or low-order polynomial) trend from the profile data. The latest magnetic data compilations for the Eurasia Basin included newer aeromagnetic data, and the shape and location of some anomalies have changed compared to the old maps (see Brozena, J. M., et al. New aerogeophysical study of the Eurasia Basin and Lomonosov Ridge: implications for basin development. Geology, 2003, 31.9: 825-828., Glebovsky, V. Yu, et al. Formation of the Eurasia Basin in the Arctic Ocean as inferred from geohistorical analysis of the anomalous magnetic field. Geotectonics, 2006, 40.4: 263-281.). In my opinion, the data quality is not sufficient for the type of analysis presented in this paper.
In the previous works, such as those cited above, the standard model oceanic crust magnetization (without patchy magnetization) was used for modeling and identification of magnetic isochrons. It is unclear how the new model performs compared to the previous models. Does it provide a better fit to the observations? Looking at Figure 2, the fit of the observed and modeled magnetic anomalies is not perfect, and sometimes the mismatch is very large. Would the identification of isochrons and finite rotations change if we consider the new crustal magnetization model? The reference for the standard workflow can be Merkouriev, S., & DeMets, C. (2014). High-resolution Neogene reconstructions of Eurasia-North America Plate motion. Geophysical Journal International, 198(1), 366-384.
The provided test for the effect of sedimentary cover is performed between the Yermak Plateau and Morris Jesup Rise in an area with very little sediments. There are seismic reflection data in the east where the sediments are well expressed, and the test in this area would be useful too. For example, see Nikishin, A. M., Gaina, C., Petrov, E. I., Malyshev, N. A., & Freiman, S. I. (2018). Eurasia Basin and Gakkel Ridge, Arctic Ocean: Crustal asymmetry, ultra-slow spreading and continental rifting revealed by new seismic data. Tectonophysics, 746, 64-82.

(Remarks on code availability)

Restricted
The record is publicly accessible, but files are restricted to users with access.

Reviewer #2

(Remarks to the Author)
General Comments: Overall I thought this was a very nice study (big kudos to utilizing 50-year-old archival aeromagnetic data!) with significant results. I was particularly impressed with Figure 3b – a simple figure that places these results in a global context, and formulates a quite elegant relationship between spreading rate and magmatic spreading fractions. I think it will hold great importance for many other researcher on mid-ocean ridge processes. The authors' magnetic cycle hypothesis of Figure 5 is straightforward and a plausible explanation for their observations of magmatic, intermediate and starved spreading modes. I don't have a lot of criticism, but I was left wondering, in the forward model/data comparison of Figure 2: what constitutes a good or bad fit? Or: how non-unique is the model? Just looking at Figure 2, the data/model comparison looked pretty good in most places, but there were others where I was puzzled as to why the authors might have included one gabbroic section or another, and the fit wasn't always convincing. I would appreciate it if the authors could speak to those issues in their revision.

Specific Comments:
Line 54: reference Figure 1 when the Gakkel Ridge is first mentioned
Line 72: reference Figure 1 again for the different zones
Line 83: change "alternance" to "alternation"
Lines 87-100: This paragraph could use some tightening to better explain the rationale for why you are using the three different magnetic layer tops. Which profiles had the seismic data?
Line 93: delete "one"
Line 94: delete "one"
Line 108: which side is the northern flank?
Lines 110-113: awkward sentence. Should this be part of the discussion?
Line 133: change "alternance" to "alternation"
Lines 141-144: Where is the interpolation? There are large gaps between profiles, especially on the right (eastern) side. Be explicit about how the different types of seafloor are established. Is this through modeling each profile as was done in Figure 2?
Line 164: delete "it should be noted that"
Figure 1: In the magnetic anomalies, is black (white) positive or negative? Green dots for peridotite are very difficult to see on colored background - especially for color blind folks like myself.
Figure 2: The details of these panels are very difficult to see on the page-width version. I had to scroll down to the enlarged version of this figure; when published, the pdf/printed version won't be so large. I suggest reformatting to a 1-column figure so that each of the panels can be page width. Need to specify which direction (negative or positive distances) is north and which is south. It would be good if the color scheme for magmatic and amagmatic spreading were the same here as in Figure 3.
Figure 3. Like Figure 2, it would be better to make this one-column, especially so that panel a) can be full-page width. Panel a) could use some improvements. The bands that display the data are odd in two ways: (1) the boundary between magmatic and amagmatic areas is at a high angle, rather than e-w, and (2) the bands get thinner to the east, such that it gets more and more difficult to distinguish between magmatic and amagmatic areas. I'm guessing these are projection issues, but I'm not certain. In any case, the bands should be a constant thickness across the figure, and the boundaries should be horizontal. Another curious issue: the figure appears to show some sort of shading but there is no explanation as to what this represents.
Figure 4: Identify what MM, MT and TT are acronyms for. Profile numbers are not useful to the reader for the x axis. Use longitude so that this figure can be compared to Figure 3 Need to mark the WZV, SZV and EZV

(Remarks on code availability)

Reviewer #3

(Remarks to the Author)
The analysis of Arctic magnetic anomalies in this manuscript reveals much important information concerning the time and space distribution of amagmatic versus magmatic crust created at ultraslow seafloor spreading centers. The results shown in Figures 1 through 4 reveal the distribution and evolution of these two modes of seafloor creation in the Arctic. I was surprised that the authors were able to classify these spreding segments through time with not two, but three categories. The obvious first two categories are (1) magmatic on both sides of the spreading center and (2) amagmatic on both sides of the spreading center. What was surprising to me was the now well-documented existence of spreading centers that were magmatic on one flank of the ridge while being amagmatic on the other flank of the ridge.

Figure 3b nicely shows that the Gakkel Ridge has the highest percentage of amagmatic seafloor (68%) of any spreading center in the world and nicely compares it with the Southwest Indian Ridge and the Mid-Atlantic Ridge.

The paper very much deserves to be published in a widely read journal.

Some minor comments:
In line 7 and elsewhere in the manuscript the word "alternances" appears, which i do not believe is a word in English. I believe the correct word in Engligh is "alternations".
In line 13 and elsewhere the word "period" is used. I believe that in geologic writing in English "Period" is best reserved for the formal divisions of geologic time using that word (e.g., the Neogene Period). It can also be used to discuss phenomena that are truly periodic. Instead I think that the word "interval" or "time interval" is generally preferable if a formal unit of geologic time is not meant.
Line 63 (and possibly elsewhere, and maybe I'm being a curmudgeon here) the correct usage in this context with "Compared with", not "Compared to".
Line 74: How thick?
Near line 92, did you consider estimating the depth to basement from the power spectra of the magnetic profiles?
Line 219: "randomly" is not very specific. Maybe you mean "uniformly randomly".

(Remarks on code availability)

Version 1:

Reviewer comments:

Reviewer #1

(Remarks to the Author)
The authors have made significant efforts to address the reviewer's comments, and the clarity of the manuscript has improved. However, I still feel that several issues remain and should be considered, together with a thorough polishing of the language and an improvement in figure quality.
The authors attempt to address the reviewer's two main concerns:
1. the uncertainty of the data relative to the analysed amplitudes, and
2. the need to demonstrate that introducing a patchy magnetization model is an alternative or superior approach compared to previous/standard models.
1. In Supplementary Figure S1, a comparison of two surveys is shown as a magnetic profile map. Three boxed areas are highlighted where the fit is considered particularly good. However, this is difficult to assess for several reasons: the two surveys are plotted using different colour schemes (the 1970s tracks in black and white, and the 1998–1999 data in blue and red), and the figure is too small to clearly evaluate the differences. A standard way to illustrate the quality of aeromagnetic data is to show the misfit at profile crossing points. This would allow us to assess both the magnitude of the errors and whether any spatial pattern exists in their distribution. In addition, total or regional RMS errors could be calculated and would provide useful information for the modelling.
2. I am still missing a figure comparing the full and patchy magnetization models. To me, the logical sequence would be to start from what was previously known or assumed and then demonstrate the improvement achieved in this study. For one or two representative profiles, show a model in which the top follows basement topography and the crust has a constant thickness of 2–4 km. Then apply a recent geomagnetic polarity timescale (e.g., Ogg 2020) and a spreading-rate history (e.g., Glebovsky et al., 2006), assuming magnetization throughout the entire crust. Compute the RMS error between the observed and modelled magnetic anomalies and analyse the distribution of the misfit. Next, use the same model geometry and spreading-rate history, but allow the magnetization to vary spatially and temporally according to the proposed patchy model. Again, compute the RMS error and analyse the misfit distribution. Finally, discuss the strengths and weaknesses of both approaches and provide arguments supporting the preferred patchy saturation model.
Other comments:
Figure S2: The polarity plot shows a strange "saw-tooth" pattern. Please correct this issue. The same problem appears in other figures. Is this a resolution issue?
Figure S3: I find it difficult to believe that the global bathymetry is represented so poorly. Did you check the IBCAO grid? In the legend, both basement and topography are shown as red lines. In some places, the topography appears below the basement surface. Please check the resolution and plotting.
Figure S4: Limit the x-axis to the range 0–0.5 cycles/m.
In the response, the isochrons modelled in this study are compared with grid contours. To me, the differences appear very large, especially for anomalies 6 and 13. The identified isochrons should instead be compared with the actual picks available from the same research group.
Figure 1: Add chron C13 and provide the appropriate reference. Grid contours are not sufficiently accurate; actual picks should be used.
Figure 2: Correct the "saw-tooth" pattern, add a legend indicating the observed and modelled magnetic anomalies, and provide RMS errors. Also indicate the assumed mean spreading rate for each segment (in the figure or in captions), together with the mean and variance of the spreading-rate perturbations introduced in the model. Add a reference to the geomagnetic polarity timescale.

(Remarks on code availability)
Please, add a readme file, make sure that all dependencies/functions are included in the archive or provide a link where one

can download them. Use relative paths.

Reviewer #2

(Remarks to the Author)
I find that the authors have satisfactorily addressed the comments in my first review, and I support publication in its present form.

John Goff
University of Texas Institute for Geophysics

(Remarks on code availability)

Version 2:

Reviewer comments:

Reviewer #1

(Remarks to the Author)
authors addressed comments in a good way.

(Remarks on code availability)
Code runs well. The readme file and example are supplied.

Open Access This Peer Review File is licensed under a Creative Commons Attribution 4.0 International License, which permits use, sharing, adaptation, distribution and reproduction in any medium or format, as long as you give appropriate credit to the original author(s) and the source, provide a link to the Creative Commons license, and indicate if changes were made.
In cases where reviewers are anonymous, credit should be given to 'Anonymous Referee' and the source.
The images or other third party material in this Peer Review File are included in the article's Creative Commons license, unless indicated otherwise in a credit line to the material. If material is not included in the article's Creative Commons license and your intended use is not permitted by statutory regulation or exceeds the permitted use, you will need to obtain permission directly from the copyright holder.
To view a copy of this license, visit https://creativecommons.org/licenses/by/4.0/

We appreciate the comments and suggestions of the reviewers to help us improve the manuscript. Comments of the reviewers are in Black and our responses are in blue.

Reviewer #1 (Remarks to the Author):

The paper addresses the variation of the magmatic activity along the ultraslow spreading Gakkel Ridge in the Arctic Ocean. The authors used analysis of historical aeromagnetic data to correlate the intensity of the magnetic anomalies with the periods of magmatic production at the spreading axis. They performed magnetic modeling of the oceanic crust from between 35 Ma and the present time on both sides of the ridge axis. The analysis addressed the intensity and symmetry of the seafloor spreading magnetic anomalies. They concluded that sequences of the robust magmatism lasted about two million years and were followed by longer phases of weak magmatism and then by the starved magmatic regime. These cycles have been suggested to reflect the dynamics of the melt segregation and transport within the thick lithosphere of the ultraslow Gakkel Ridge. According to their estimates, 68% of the oceanic crust in the Eurasia Basin was formed in the starved magmatic accretion regime. The magnetic modeling was performed on the airborne magnetic profiles collected in the middle of the 70s by the U.S. NRL. Without satellite navigation at that time, it was not uncommon for airborne data collected to suffer from navigation problems. Therefore, some anomalies in old surveys can be shifted or be narrower or wider than they are due to poorly controlled positioning and speed. Another problem with magnetic data in the Arctic is the strong diurnal variation of the geomagnetic field. The geomagnetic storms and ionospheric currents can produce short-period variations of the geomagnetic field undistinguishable from geological anomalies. For aeromagnetic surveys, we just hope that the aircraft has a high enough speed such that the geomagnetic variation would be recorded as a linear trend. The standard processing would include data from the magnetic reference stations and tie-line levelling. In the Arctic Ocean, the use of base stations close enough to the survey area is logistically difficult. Therefore, we rely mostly on the levelling procedures using data from the crossing points with tie lines. The residuals at the crossing points with tie lines are used to determine and remove a linear (or low-order polynomial) trend from the profile data. The latest magnetic data compilations for the Eurasia Basin included newer aeromagnetic data, and the shape and location of some anomalies have changed compared to the old maps (see Brozena, J. M., et al. New aerogeophysical study of the Eurasia Basin and Lomonosov Ridge: implications for basin development. Geology, 2003, 31.9: 825-828., Glebovsky, V. Yu, et al. Formation of the Eurasia Basin in the Arctic Ocean as inferred from geohistorical analysis of the anomalous magnetic field. Geotectonics, 2006, 40.4: 263-281.). In my opinion, the data quality is not sufficient for the type of analysis presented in this paper.

The reviewer is concerned about the navigation of the old aeromagnetic data in the Arctic regions. Indeed, an adequate data quality is required for further analysis. The dataset used in our study was obtained from the NCEI (ex-NGDC) GEODAS database. It has been processed and corrected by researchers from the Naval Research Laboratory before being uploaded to this database, and we are quite confident that this US Navy-related lab was benefitting the best navigational technologies of the time and the best experts to process and correct the data for any bias. Corrections applied to the magnetic data include shifting, cross-correlation leveling, filtering, and IGRF corrections (as indicated in the Readme file given in the database). Navigation of the old data was handled and calibrated using the Thule_1992 dataset collected with differential GPS navigation. Any potential bias was therefore considered and corrected by leveling and filtering before the data were included in the database in November 1993. After extracting the data from the NCEI database, we only

removed a linear trend of the data to obtain clearer contrast. We compared the data used in our study with a more recent dataset acquired by the US Navy in 1998 and

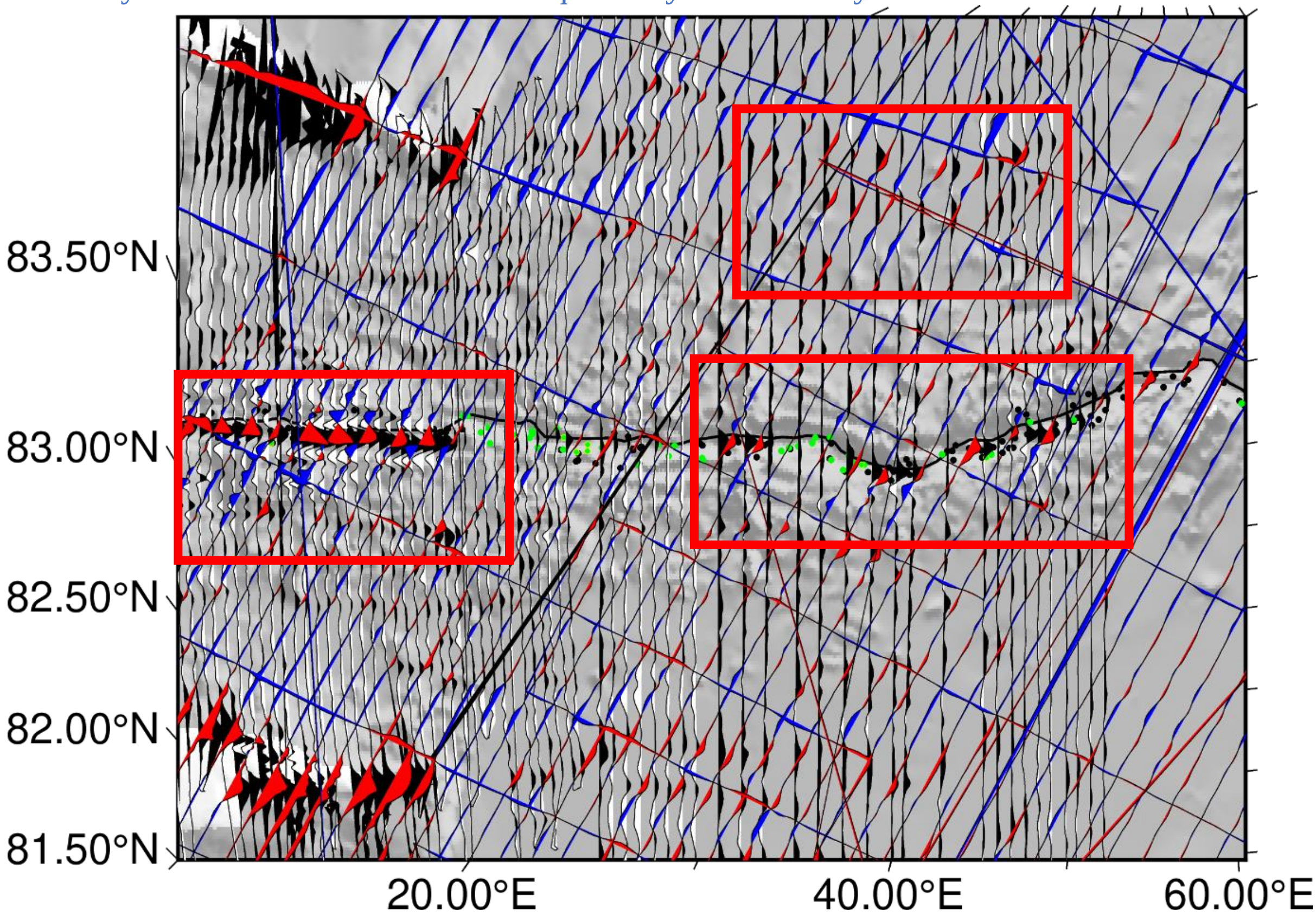

Figure 1. Comparison of the aeromagnetic datasets collected in 1970s (black and white wiggles) and 1990s (red and blue patterns). The solid black line marks the location of the Gakkel Ridge axis. The black and green dots show the locations of basalt and peridotite, respectively. The red boxes delineate the areas that clearly show the consistency between the two datasets.

1999. The crossing points of the two datasets correlate well both in the axial and off-axial areas (Red boxes in Figure 1 above). The central magnetic anomaly is over the ridge axis - strong over the West Volcanic Zone, subdued or missing in the Sparsely Volcanic Zone, and occasionally seen in the Eastern Volcanic Zone- which is consistent with the sampled rock types along the ridge axis. Off-axis, strong magnetic anomalies are depicted over the Yermak Plateau and the Morris Jesup Rise. The navigation and amplitude variation of the dataset both along and across the ridge axis appear to be sufficient to investigate the variations of magmatism. We added the validation presented above in the *Supplementary Information*

The geomagnetic storms and ionospheric currents mentioned by the reviewer do not appear in the dataset. Their influence would be characterized by large deviations from the baseline and systematic short-wavelength signals that are not seen in our data. Data collected during magnetically unquiet periods have been removed before the data were uploaded to the database.

In the previous works, such as those cited above, the standard model oceanic crust magnetization (without patchy magnetization) was used for modeling and identification of magnetic isochrons. It is unclear how the new model performs compared to the previous models. Does it provide a better fit to the observations? Looking at Figure 2, the fit of the

observed and modeled magnetic anomalies is not perfect, and sometimes the mismatch is very large. Would the identification of isochrons and finite rotations change if we consider the new crustal magnetization model? The reference for the standard workflow can be Merkouriev, S., & DeMets, C. (2014). High-resolution Neogene reconstructions of Eurasia-North America Plate motion. Geophysical Journal International, 198(1), 366-284.

The provided test for the effect of sedimentary cover is performed between the Yermak Plateau and Morris Jesup Rise in an area with very little sediments. There are seismic reflection data in the east where the sediments are well expressed, and the test in this area would be useful too. For example, see Nikishin, A. M., Gaina, C., Petrov, E. I., Malyshev, N. A., & Freiman, S. I. (2018). Eurasia Basin and Gakkel Ridge, Arctic Ocean: Crustal asymmetry, ultra-slow spreading and continental rifting revealed by new seismic data. Tectonophysics, 746, 64-82.

Our study does NOT aim to revise the well-studied isochrons and plate motions as given by the references listed by the reviewer. Instead, we took advantage of the varying magnetic anomaly amplitude over the magmatic and amagmatic seafloor to determine their distribution and in turn constrain the variation of magmatism. Our strategy is to build models based on the seafloor age, initially assuming symmetrical spreading with half spreading rates from the plate motion models, then slightly adjusting the half spreading rate to fit the main observed anomalies. Our modelling can therefore be defined as a process-oriented modelling, using a known spreading rate and assigning three different types of magnetic sources (magmatic, amagmatic with gabbro bodies, amagmatic without gabbro bodies) at each time step. If some synthetic anomalies show a significant shift with respect to the observed anomalies, we adjust the location of the reversals in the corresponding time intervals - of course in a consistent way for both conjugate flanks to conserve the full spreading rate. The amplitude of the anomalies determines the choice of magmatic or amagmatic seafloor, with or without gabbro bodies. The final isochrons retained by our model do not differ much from the initial ones derived from the plate motion models because we use and do not question the isochron identifications underlying the plate motion models. In Figure 2 below, we compare the isochrons retained by our study with those compiled for the global age map of the seafloor (Müller et al., 2019). The two sets of isochrons show only minor differences. So, our work does not challenge the existing identification of magnetic isochrons nor the finite rotations describing the Arctic plate kinematics, but may help to refine them in future works.

Simulating magnetic anomalies and identifying isochrons at ultraslow spreading ridges is always a difficult task because some anomalies are missing or merged as a consequence of the ultraslow rate and the complexity of the oceanic crust formed at these rates, with outcropping mantle and deep crustal rocks exhumed on the footwall of detachment faults. Additionally, the frequent occurrence of oceanic core complexes (OCCs) induces asymmetric spreading. As a result, because nature is complex and our knowledge of the Arctic oceanic lithosphere is still very limited, we admit that perfect models cannot be built. Some slight lateral shifts and amplitude discrepancies between the observed and synthetic anomalies have no consequence for our goal, identifying the modes of seafloor spreading and their distribution over the Eurasian Basin. We tried to fit the prominent anomalies as well as we could, which in turns allowed us to identify where and when magmatic seafloor was formed, but we do not intent to provide a perfect fit of the anomalies that may require some too complex models and, sometime, unrealistic parameters. Our choice of considering three contrasted types of magnetic sources allows recovering most of the observed anomalies, hence providing a reliable estimate of the mode of seafloor, especially on the sedimented off-

axis areas. We completed the Results section with explanations about the goals and limitations of our modelling in Lines 128-179.

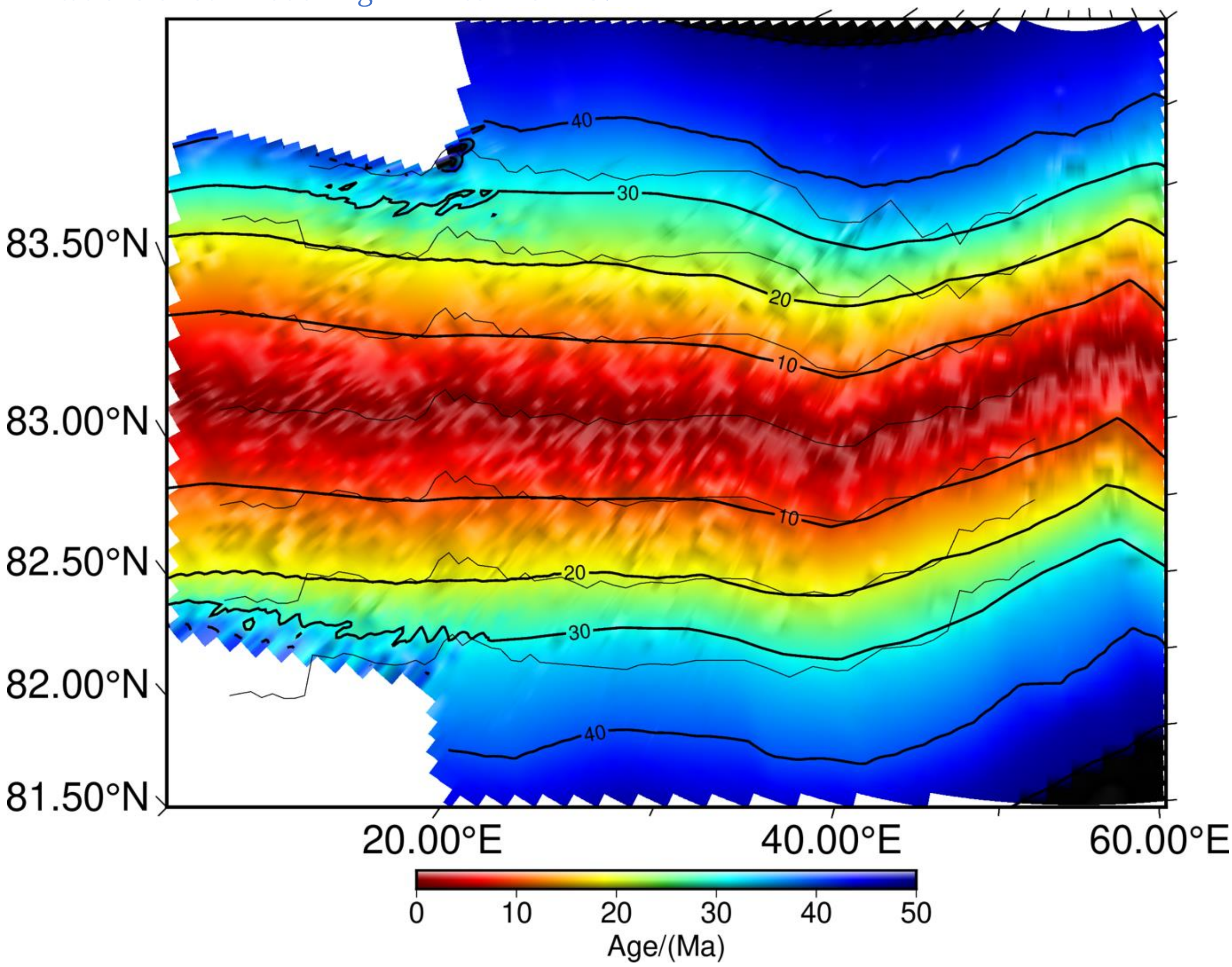

Figure 2. Comparison of the isochrons modeled in our study (thin black lines) and from Müller et al. (2019) (thick black lines).

As suggested by the reviewer, we obtained sediment thickness data along seismic profiles from Tan et al. (JGR-SE, 2024) (Figure 3 below). The seismic profile location does generally not align with that, perpendicular to the strike of the ridge axis, of our magnetic data. Only their Line 1 in the west runs along one of our magnetic profiles. The sediment thickness along this profile is generally less than 1km in the axial area. However, in some areas (e.g., 40-120 km and 160-200 km in Supplementary Information Figure 3b) we observe areas where the basement and seafloor topographies differ for up to ~1.5 km and the corresponding forward-modelled magnetic anomalies exhibit only little differences. This test clearly shows that using the basement topography in the forward modelling would not generate significant improvement on the synthetic anomalies computed at airborne altitudes, i.e., 4000 m above the seafloor. Following the suggestion of Reviewer #3, we also conducted a spectral analysis. In the short-wavelength domain (<100 m), the synthetic anomalies calculated for different top boundaries of the magnetic source layer are nearly identical, indicating that the short-wavelength signal generated by the basement topography has been filtered by the altitude of the observation datum plane above the source layer.

REDACTED

Figure 3. Location of the seismic profiles in the Eurasian basin (From Tan et al., 2024).

Specific comments:

Line 7: Language problem of the “Alternances”

We corrected it to “alternation”.

Line 9: Add “oceanic basement” after “blanketed”

We added accordingly.

Line 10: Replace “spanning” by “covering”; Change “over” to “oceanic crust formed at”

We modified accordingly.

Line 12: Add “Eurasia” before “basin”

We added Eurasian accordingly.

Line 13: Add “for the last 35 Ma” after “seafloor”.

We added it accordingly.

Line 15: Add “presumably” after the “lithosphere”

We added it accordingly.

Line 16: Add “may” before the “prevent”

We added it accordingly.

Line 17 Change “direct” to “indirect”.

We changed it accordingly.

Line 19: Change "well beyond" to "outside"
We changed to "away from"
Line 27: Add reference after the "structure".
We added the references Cannat et al., 1995, 2019 accordingly.
Line 28: Rephrasing this sentence. Change the position of "magma chambers"
We changed the sentence to "Segments with thick crust reflect robust focused magmatism and hence the occurrence of long-lived magma chambers. Segments dominated by tectonics may expose mantle rocks, hence reflecting starved magmatism lacking stable magma chamber" as the reviewer suggested.
Line 48: What about superposition and diffusion of boundaries of MA? What if one flank is sedimented?
The ultraslow spreading rate and slow cooling rate of the intrusive rocks exhumed by the detachment faults at ultraslow spreading ridges may cause the coalescence of magnetic anomalies for periods with frequent geomagnetic polarity reversals. This is the cause of some broad anomalies in intervals like C5n-C5ACn in Figure 2a, 2c (revised manuscript) or C6AAn-C6Cn in Figure 2b (revised manuscript). This "sequence effect" (Dyment et al., 1994) may reduce or enhance the amplitude of some anomalies in a rather random way, depending on the relative distance between magnetized structures and therefore on slight, local variations of the spreading rate and spreading asymmetry. It may occasionally affect our determination of the seafloor type, but the geomagnetic polarity time scale is now well known for the considered period and the spreading rate is also rather well constrained, so its impact on the distribution of magmatic and amagmatic seafloor is local and limited. If such an effect had a significant impact, then the distribution of amagmatic seafloor should be overestimated for the periods with frequent reversals such as C3n and between C6n and C9n, and the distribution of magmatic seafloor overestimated for periods with less frequent reversals. We examined the statistical distribution of amagmatic seafloor for different time intervals and found no bias toward more amagmatic seafloor at any time interval. Furthermore, the resolution of our study makes unlikely any significant impact of the "sequence effect" on our regional classification between magmatic and amagmatic seafloor from aeromagnetic data. Indeed, the spreading rate is too slow to make variations within ~1 Ma clearly reflected on the data as. The superposition and diffusion of boundaries, understood as reflecting the piling of lava flows in the extrusive basalt and the slow cooling of intrusive gabbro bodies, are not observable 4000 m above the magnetized sources. We do observe them in studies using AUV and high-resolution shipboard magnetic data, for example over some specific OCCs (e.g., Zhou et al., 2022, Geology; Dyment et al., EPSL, in revision), but they are not significant at the scale of the present study.
Another test displayed in our Supplementary Information confirms the negligible influence of sediments on the aeromagnetic data over the Gakkel Ridge with >4000 m water depth.

Line 49-50: use consistent words to describe the states of magmatism
We kept them consistent to be "robust, intermediate, and starved" in the revised manuscript.
Line 58: Change "undoubtedly confirmed" to "inferred from"
We changed it accordingly
Line 60: Remove "different lithospheric thickness"
Geophysical studies along the Gakkel Ridge found different lithospheric thickness at the WVZ, SMZ, and EVZ, where the most amagmatic segment SMZ shows a rather deep micro-seismicity (Schlindwein and Schmid, 2016, Nature). This is an important geophysical evidence for the segmentation of the Gakkel Ridge. We choose to keep this relevant information as complementing the different "dredged rock samples" at the end of sentence.

Line 77-78: Remove "further demonstrating the ability of marine magnetic anomaly to discriminate seafloor types".
We removed it accordingly
Line 80-81: "~10 Ma and disappear over older regions in the WVZ. Conversely, the SMZ shows no magnetic anomaly within the recent -20 M" This is not true. LMA are mapped there.
There are some anomalies within the SMZ, but very subdued and discontinuous as the magnetic maps of Gaina et al. (2014) and Brozena et al. (2003) illustrated. We described it more cautiously by using "subdued and discontinuous".
Line 92: Add "profiles" after the "reflection"
We added it accordingly
Line 98: change to "aeromagnetic observation plane"
We changed it accordingly
Line 102: What do you mean the "representative"?
We selected the profiles that can represent the different states of magmatism in the three segments. We rephrased the sentence to be clearer. It now reads as:
"We selected three profiles as case studies that show the varying states of magmatism in time and along the ridge axis over the three major segments of the Gakkel Ridge".
Line 112: "why older crust displays stronger anomaly than the younger one". The influence of superposition? Compare full and patchy magnetization models.
We refer the reviewer to our previous reply about the superposition effect: again, if it has a significant impact, we should observe a bias in the distribution of magmatic and amagmatic seafloor on some specific time intervals, that is not observed. We therefore interpret such variations as reflecting variations in the source, more specifically in the magmatic or amagmatic type of seafloor.

We added description of the magnetization profile shown in Figure 2b (revised manuscript) and compared it with the full magnetization model of Figure 2a (revised manuscript) as follows:
"A profile in the EVZ exhibits similar features. The central magnetic anomaly spreads over ~3 Ma but displays an amplitude ~200 nT lower than does the same anomaly on the profile in the WVZ discussed above. Off-axis, the magmatic seafloor appears sparsely for time intervals C5An-C5Cn, C6Cn-C8n, C10n-C12n, and C18n-C19n on the southern flank and C5Cn, C6AAn-C6Cn, C10n-C12n, and C16n-C19n on the northern flank. Before C6Cn (~24 Ma), magmatic crust was more abundant and generated stronger magnetic anomalies, while sparser and weaker magnetic anomalies are observed from C6Cn to present. The alternation of magmatic and amagmatic seafloor spreading is faster and the duration of magmatically robust episodes is shorter for this period. A possible reason is that the slight ridge obliquity and slower full spreading rate in this eastern area restricts the effective magma supply, shortening the magmatic episodes[34]. The faster magmatic variations in this segment induce a more complex crustal structure and make difficult a perfect fit of our model for each anomaly, especially off-axis where information on the age, spreading rate, and local tectonic history is limited and geological parameters such as the degree of basalt alteration, crustal faulting, ... are poorly constrained. However, even imperfect, our modeling aims at identifying regionally the types of seafloor by primarily relying on the amplitude of prominent magnetic anomalies."

Line 133: Change "alternance"
We changed it to "alternation" accordingly.

Line 153: “from 14 km/Myr west of WVZ to 10 km/Myr east of the EVZ”. What is predicted spreading rate using Euler poles and distance?

The spreading rate predicted by plate motion models is 13-11 km/Myr in this area. In our model, we adjust the seafloor spreading rate in the different periods to better fit the observed magnetic anomalies. Such short-term spreading rate variations are common along the mid-ocean ridge and may be caused by the occurrence of detachment fault or ridge propagation. Due to such uncertainties, the modeled spreading rate may slightly differ from that predicted by the plate motion models. We added the spreading rate calculated from the finite rotation Euler pole of plate reconstructions by previous studies.

“The modelled profiles also encompass this spreading rate variation, ranging from ~14 km/Myr west of WVZ to ~10 km/Myr east of the EVZ, in general agreement with the predicted spreading rate range of 13-11 km/Myr derived from plate tectonic models.”

Line 185: What about ridges orthogonal to the spreading axis?

Line 194-195: “roughly spanning 2Myr, is always followed by a phase of intermediate magmatism” Again, what about orthogonal ridges.

We think the orthogonal ridges that the reviewer refers to are the ones marked in the figure below. They are not symmetric on the two flanks adjoining the ridge axis and do not offset the ridge. They are only seen in the axial region but show no off-axis trace on the bathymetric map. No corresponding signature are observed either in the gravity or the magnetic map (Figure below). Therefore, we do not think they can be interpreted as transform faults or fracture zones. These interesting features may be the result of different mechanisms. For instance, a detachment fault may exhume mantle rocks within a corridor parallel to the spreading direction and elevate the terrain in the footwall. Alternatively, the existence of narrow magmatic centers may also create thicker crust and shallow topography within similar corridors. Without high-resolution bathymetry, geophysical data, and dredge rock samples, we cannot infer much about their formation mechanism. As they do not offset the spreading axis, we do not expect them to significantly affect the location of magmatism within the lithosphere at a regional scale.

Two magnetic profiles in our dataset cross the two western orthogonal ridges marked as below, i.e., Profiles 54 and 60 shown in Figure 4 of the manuscript. We observe narrow windows with strong magmatism over the last 5 Ma in these two areas. The magmatic seafloor occurs on both flanks generating strong magnetic anomalies, which is consistent with the observations of Jokat et al. (2003) who discarded these ridges as transform fault but interpreted them as formed by trapped melt in the mantle. From this limited analysis, we would also favor the interpretation of strong localized magmatism within narrow corridors as the cause of these orthogonal ridges.

REDACTED

Figure 4. Bathymetry (top), gravity (bottom left) and magnetic (bottom right) maps of the Gakkel Ridge (From Brozena et al., 2003). The black boxes mark the locations of the orthogonal ridges.

Line 215: What means of “mantle probably restored”

According to Zhang et al., (2024, Nature), active mantle upwelling maybe a possible mechanism to explain the focused strong magmatism observed in the eastern Gakkel Ridge. We here want to express that the active mantle upwelling happened in the past resulted in the

magmatic bursts previously, but do not exist anymore beneath the current axial region where the magmatism is weak. The sentence was not very clear and we rephrased it as below: "Whether any similar active mantle upwelling existed beneath the past magmatic bursts shown by our magnetic study cannot be proven because the mantle dynamics keep changing and the current state of the melting zone beneath the ridge axis cannot represent the past."

Line 226: Where is the ancient recycled lithospheric mantle?
We agree with the reviewer that our original wording may have unintentionally implied a specific spatial location beneath the ridge axis.
In fact, previous studies do not suggest that such ancient refractory mantle forms any discrete or spatially continuous body directly beneath specific locations along the ridge. Instead, geochemical and isotopic evidence indicates that the mantle beneath the Gakkel Ridge is highly heterogeneous, containing distributed refractory domains formed by ancient melt extraction events (e.g., Liu et al., 2008; Salters et al., 2011). These refractory components are typically not directly sampled by MORB melts due to their low melt productivity, and therefore their presence is inferred from integrated geochemical signatures rather than spatially resolved observations. In Figure 5, we also show that when the refractory component in the melting zone is dominant, then the melt productivity is limited resulting in the amagmatic seafloor spreading. We reword the sentence as below:
"Geochemical isotopic composition of the rock samples such as the Re-Os of ultramafic peridotite and Hf-Nd of basalt indicates the existence of ancient recycled lithospheric mantle component within a heterogeneous mantle system beneath the axis of the Gakkel Ridge."

Line 247: Change to "temporal variation of magmatism".
We changed it accordingly.
Line 265: add "airborne"
We added it accordingly
Line 271-272: The data had problems with navigation and diurnal correction that affects the shape of the anomalies.
See our reply above to this concern of the reviewer. We added a description of the processing and corrections done prior the data were released for clarity, as below:
"Navigation, cross-correlation leveling, filtering, and IGRF corrections were previously applied (Supplementary Information) to overcome any navigation problem and influence of external fields. We only removed long-wavelengths trends from the archived data. The corrected magnetic anomaly correlates well with a new dataset collected in 1998-1999 with GPS navigation. Both the location and amplitude of the magnetic anomaly at crossing points of the two independent datasets are consistent. Then we conducted the modeling by using the method of ref [17,18] for 60 profiles."

Figure 2: show identified isochrons. Show full and patchy models next to each other.
We modified the figure as the reviewer suggested. We also follow the suggestion of the Reviewer #2 to put the three panels in one column to make them shown in full page.
Figure 3a: reference of the age?
We added the references of the age in the figure caption.

Remarks on code availability:
Restricted
The record is publicly accessible, but files are restricted to users with access.
We made them open-access now

Reviewer #2 (Remarks to the Author):

General Comments: Overall I thought this was a very nice study (big kudos to utilizing 50-year-old archival aeromagnetic data!) with significant results. I was particularly impressed with Figure 3b – a simple figure that places these results in a global context, and formulates a quite elegant relationship between spreading rate and magmatic spreading fractions. I think it will hold great importance for many other researchers on mid-ocean ridge processes. The authors' magnetic cycle hypothesis of Figure 5 is straightforward and a plausible explanation for their observations of magmatic, intermediate and starved spreading modes. I don't have a lot of criticism, but I was left wondering, in the forward model/data comparison of Figure 2: what constitutes a good or bad fit? Or: how non-unique is the model? Just looking at Figure 2, the data/model comparison looked pretty good in most places, but there were others where I was puzzled as to why the authors might have included one gabbroic section or another, and the fit wasn't always convincing. I would appreciate it if the authors could speak to those issues in their revision.
In our modelling exercise, we tried to fit the prominent magnetic anomalies with reasonable parameters and assumptions like the spreading rate, crustal structure, and equivalent magnetization to distinguish the magmatic and amagmatic seafloor in a regional perspective. The more prior information we have, the better is the fit that can be achieved. For example, in the WVZ (Figure 2a in manuscript) and EVZ (Figure 2b in manuscript) where the central magnetic anomaly is clearly seen at in the ridge axis, we can achieve a better fit for the models within the last 10 Ma. Off-axis, the alteration of basalt reduces its magnetization, the crust may be faulted, the spreading rate may have varied in the past, etc... are all possible influences on our models. We know that there should be magmatic seafloor off-axis (i.e., Figure 2c) because high-amplitude magnetic anomalies are observed there with even stronger amplitudes than in younger areas. Conversely, we know that magmatism is starved at the axis of the SMZ, a point confirmed by the lack of a well-defined central magnetic anomaly there (Figure 2c). One difficulty is that we do not know exactly where the zero-age is located in this area. If the plate separation is accommodated by "flip-flop" detachment faults (Sauter et al., 2013), then the ridge axis frequently jumps back and forth. We tried to set up the zero-age to fit most of the anomalies, but there are still some synthetic anomalies slightly shifted laterally with respect to the observed anomalies. Furthermore, the occurrence of detachment faults results in asymmetric spreading, accelerating the half spreading rate on one flank within a short period (i.e., <2 Myr). This could also affect our model fitting. However, we cannot rule out this effect by considering the occurrence of detachment faults without knowing their precise location.

Simulating the magnetic anomalies and identifying the isochrons at ultraslow spreading ridges is always challenging because some anomalies are missing or features are merged due to ultraslow spreading rate and the occurrence of the mantle rocks exhumed by the detachment fault that may induce asymmetric spreading as well. We admit that we cannot build a perfect model, because the nature is complex and our knowledge of the Arctic seafloor is too limited. So sometimes a certain amount of adjustment (lateral shift) should be allowed and amplitude discrepancy between the synthetic and observed anomalies is to be expected. We tried to fit the most prominent anomalies as well as possible, which makes possible classifying where and when magmatic and amagmatic seafloor was formed, but we did certainly not target a perfect fit of the anomalies with unrealistic model parameters. Our study considers contrasted magnetic sources to explain the observed anomalies, and provides a first-order estimation of the types of seafloor especially for sedimented off-axis areas.

As for the gabbroic bodies, we added them to recover some small magnetic anomalies within amagmatic spreading period. Such anomalies are generally narrow and of low amplitude. A magmatic crust with a basalt layer would generate strong anomalies which do not fit them. We therefore added these small gabbroic bodies to achieve a better fit. These intrusive gabbro bodies bear slightly stronger magnetization than the gabbro layer in the magmatic crust to take into account higher fractionation degree in the shallow crust. However, small volcanic patches with random magnetization vector rafted by the detachment fault could also generate these small magnetic anomalies within the amagmatic spreading period. Without dredges or drilling, we cannot define their nature. They were only used to better fit the small anomalies but do not affect the distinction between magmatic and amagmatic seafloor relying on the prominent anomalies.

In summary, our model aims at a regional classification of magmatic and amagmatic seafloor and their distribution. Our model is certainly not unique as we face the intrinsic non-uniqueness of the potential field data. The thickness of the layers, their magnetization, and the crustal structure, imposed in our models, are probably more variable. Nevertheless, the contrasted magnetic anomalies correspond to different types seafloor at the ultraslow spreading ridges and our physically possible models help determining the lateral distribution of these types of seafloor. We described the limitations and strategies during the model construction more in the Result section:

Lines 128-130

"Gabbro intrusions or small volcanic patches bearing moderate magnetization (see Material and method) within the amagmatic lithosphere are used to model small magnetic anomalies that are too narrow and weak to be due to magmatic seafloor."

Lines 133-139

"Observed magnetic anomalies do not only reflect the geomagnetic polarity interval they are located on but integrate the effect of adjacent polarity intervals as well into broader, less resolved anomalies. This consequence of convolving the geomagnetic polarity time scale with a punctual magnetic source model (e.g., Schouten and McCamy, 1972) is an inevitable difficulty for magnetic anomaly modeling at ultraslow spreading ridge, for which a resolution better than 1 Myr cannot be reached."

Lines 161-167

"The faster magmatic variations in this segment induce a more complex crustal structure and make difficult a perfect fit of our model for each anomaly, especially off-axis where information on the age, spreading rate, and local tectonic history is limited and geological parameters such as the degree of basalt alteration, crustal faulting, ... are poorly constrained. However, even imperfect, our modeling aims at identifying regionally the types of seafloor by primarily relying on the amplitude of prominent magnetic anomalies."

Lines 172-179

"Long weakly magmatic or amagmatic episodes increase the difficulty to fit models to the magnetic anomalies. Possible existence of detachment faults may induce asymmetric spreading in short time windows (<2Myr), but in the long term the seafloor age does not depart much from a general symmetry on the two flanks of the ridge[2]. Additionally, the zero-age point of the model is hard to set without reference of the central magnetic anomaly, resulting in the synthetic anomaly being slightly shifted laterally, although having negligible effects on identifying the types of seafloor regionally."

Specific Comments:

Line 54: reference Figure 1 when the Gakkel Ridge is first mentioned

We added the reference to Figure 1 accordingly.
Line 72: reference Figure 1 again for the different zones
We added the reference to Figure 1 accordingly.
Line 83: change “alternance” to “alternation”
We changed it accordingly.
Lines 87-100: This paragraph could use some tightening to better explain the rationale for why you are using the three different magnetic layer tops. Which profiles had the seismic data?
We conducted this forward modeling using different tops for the magnetic layer to test the influence of the sediment on the magnetic anomalies. As Figure S2 shows in the Supplementary Information, a seismic profile in the west of WVZ has the same location as one of our magnetic profiles. We calculated the synthetic magnetic anomalies by using magnetic layer topped by the topography extracted from global bathymetry grid data, shipboard bathymetry survey, and the real basement derived from seismic reflection. The results show little influence of the sediment thickness at the altitude of the aeromagnetic data and using the seafloor as the top of the magnetized layer is therefore an acceptable simplification.

We added a subtitle and rephrased this paragraph as the reviewer suggested.
“**Influence of sediment thickness**

To quantitatively evaluate the spatiotemporal variation of magmatism, we conducted forward modeling on each profile applying the method of ref[17,18]. However, the thick sediment blanketed on the seabed hides the basement topography which inherently affects the recorded magnetic field. To evaluate the impact of the sediment thickness that may reach up to 1000 m[30], we built a forward model by using different top boundaries of the magnetic layer: one is the basement topography underneath the varying sediment thickness obtained from seismic reflection profiles[31], another is the seafloor topography surveyed by the shipboard echosounder along the same seismic profile, and the last is the seafloor topography extracted from the Global Multi-Resolution Topography[32]. The location of these profiles is shown in *Supplementary Information* Figure S3. The difference between these three top boundaries is bigger when the sediment is thicker, allowing to test the influence of varying sediment thickness on the observed magnetic anomaly. The dominant effect on the modelled anomalies is the contrast between the strongly magnetic magmatic seafloor and the non-magnetic amagmatic one. Differences in the geometry of the top boundary of the magnetic layer only result in negligible variations of the modelled anomaly, which is due to the large distance between the magnetic source and the aeromagnetic observation plane (>4 km bathymetry, 300 m flying altitude, and <1 km sedimentary thickness), in turn smoothing the short-wavelength magnetic signal (Supplementary Information). This test illustrates that the sediment thickness has only a minor influence on the recorded aeromagnetic data, and suggests that magnetic models based on the seafloor topography allow to constrain the type of the crust beneath the sediment.”

Line 93: delete “one”
We removed it accordingly
Line 94: delete “one”
We removed it accordingly
Line 108: which side is the northern flank?
We set the northern flank to positive distance in our model. The reviewer is correct, we should mark it in the Figure 2. We clarified it in the Figure 2.

Lines 110-113: awkward sentence. Should this be part of the discussion?
Our Discussion sections focus on the mechanism of the episodic melt burst but not on the individual profile described in the result section, so we chose to rephrase the sentence as follows:
"The large amplitude variation of these magnetic anomaly is inconsistent with the observed topography and frequent geomagnetic magnetic reversals if a uniform magnetized source is considered. Instead, amagmatic seafloor with gabbro intrusions or small volcanic patches during the C3n-C4n interval and a short magmatic seafloor spreading event during the C4n explain well these observations."

Line 133: change "alternance" to "alternation"
We changed it accordingly
Lines 141-144: Where is the interpolation? There are large gaps between profiles, especially on the right (eastern) side. Be explicit about how the different types of seafloor are established. Is this through modeling each profile as was done in Figure 2?
We rephrased these sentences as the reviewer suggested to avoid confusion.
"We located the different types of seafloor derived from the modeling of each profile in map view to unravel their geographical distribution. For regional characterization, we interpolated the points sampled along profiles onto a grid and used values of 1 and 0 to indicate magmatic and amagmatic seafloor, respectively, whereas the gaps between the profiles were filled by NaN value. The ratio of magmatic and amagmatic seafloor were then calculated, showing that amagmatic seafloor accounts for 68% of the total seafloor in contrast to 32% for the magmatic seafloor, characterizing the seafloor spreading history in the Arctic Ocean for the last ~35 Ma (Figure 3)."

Line 164: delete "it should be noted that"
We removed it accordingly.
Figure 1: In the magnetic anomalies, is black (white) positive or negative? Green dots for peridotite are very difficult to see on colored background - especially for color blind folks like myself.
The black is positive and white is negative. We added this information in the caption. The reviewer is correct, and we changed the colors of the dots.
Figure 2: The details of these panels are very difficult to see on the page-width version. I had to scroll down to the enlarged version of this figure; when published, the pdf/printed version won't be so large. I suggest reformatting to a 1-column figure so that each of the panels can be page width. Need to specify which direction (negative or positive distances) is north and which is south. It would be good if the color scheme for magmatic and amagmatic spreading were the same here as in Figure 3.
We modified the figure as the reviewer suggested. Put panels into one column, defined the north and south direction, and used the same color of magmatic and amagmatic seafloor as Figure 3.
Figure 3. Like Figure 2, it would be better to make this one-column, especially so that panel a) can be full-page width. Panel a) could use some improvements. The bands that display the data are odd in two ways: (1) the boundary between magmatic and amagmatic areas is at a high angle, rather than e-w, and (2) the bands get thinner to the east, such that it gets more and more difficult to distinguish between magmatic and amagmatic areas. I'm guessing these are projection issues, but I'm not certain. In any case, the bands should be a constant thickness across the figure, and the boundaries should be horizontal. Another curious issue: the figure appears to show some sort of shading but there is no explanation as to what this represents.

We modified the figure as the reviewer suggested. We put the two panels into one column. For the panel a), the boundary between the magmatic and amagmatic areas have been adjusted to be horizontal but not strictly in E-W. The specific projection in polar area would skew some shapes for visualization. That is why the eastern profiles seems to be narrower especially close the pole. We set up a constant width for the profiles but the area close to the pole still shows some skewness. The shading of the bathymetry has been removed because it does not provide any necessary information.

Figure 4: Identify what MM, MT and TT are acronyms for. Profile numbers are not useful to the reader for the x axis. Use longitude so that this figure can be compared to Figure 3. Need to mark the WZV, SZV and EZV.

We modified the figure and defined the MM, MT, and TT in the caption, changed the profile numbers to longitude, and marked WVZ, SVZ, and EVZ as the reviewer suggested.

Reviewer #3 (Remarks to the Author):

The analysis of Arctic magnetic anomalies in this manuscript reveals much important information concerning the time and space distribution of amagmatic versus magmatic crust created at ultraslow seafloor spreading centers. The results shown in Figures 1 through 4 reveal the distribution and evolution of these two modes of seafloor creation in the Arctic. I was surprised that the authors were able to classify these spreading segments through time with not two, but three categories. The obvious first two categories are (1) magmatic on both sides of the spreading center and (2) amagmatic on both sides of the spreading center. What was surprising to me was the now well-documented existence of spreading centers that were magmatic on one flank of the ridge while being amagmatic on the other flank of the ridge.

Figure 3b nicely shows that the Gakkel Ridge has the highest percentage of amagmatic seafloor (68%) of any spreading center in the world and nicely compares it with the Southwest Indian Ridge and the Mid-Atlantic Ridge.

The paper very much deserves to be published in a widely read journal.

Some minor comments:

In line 7 and elsewhere in the manuscript the word "alternances" appears, which i do not believe is a word in English. I believe the correct word in Engligh is "alternations".

The reviewer is correct. We corrected all the “alternance” to “alternations”.

In line 13 and elsewhere the word "period" is used. I believe that in geologic writing in English "Period" is best reserved for the formal divisions of geologic time using that word (e.g., the Neogene Period). It can also be used to discuss phenomena that are truly periodic. Instead I think that the word "interval" or "time interval" is generally preferable if a formal unit of geologic time is not meant.

When possible, we replaced "period" by "time interval", but this formulation is sometimes heavy and "period" is also a common word in English meaning exactly that: a "time interval". So, we kept "period" when it was lighter and without ambiguity.

Line 63 (and possibly elsewhere, and maybe I'm being a curmudgeon here) the correct usage in this context with "Compared with", not "Compared to".

We changed it accordingly (two occurrences in text).

Line 74: How thick?

According to a recent gravity study, the inverted crustal thickness in the WVZ is ~6 km whereas 2-4 km in the SMZ. We added this information in the text.

Near line 92, did you consider estimating the depth to basement from the power spectra of the magnetic profiles?

We had not done the power spectra analysis of the magnetic anomalies calculated from different top boundaries of the magnetic layer, but did it as suggested by the reviewer. The results shows that the three synthetic magnetic anomalies calculated using different top boundaries of the magnetic layer only exhibit minor differences in the spatial domain, with only long-wavelength (>100 m) content no matter whether the amplitude spectrum, phase spectrum, or power spectral density plots are used. As for short-wavelengths (<100 m), the three datasets are nearly identical, indicating that the short-wavelength signals generated by the basement topography have been filtered at the high altitude of the observation datum plane above the source layer. We added this analysis in the Supplementary Information.

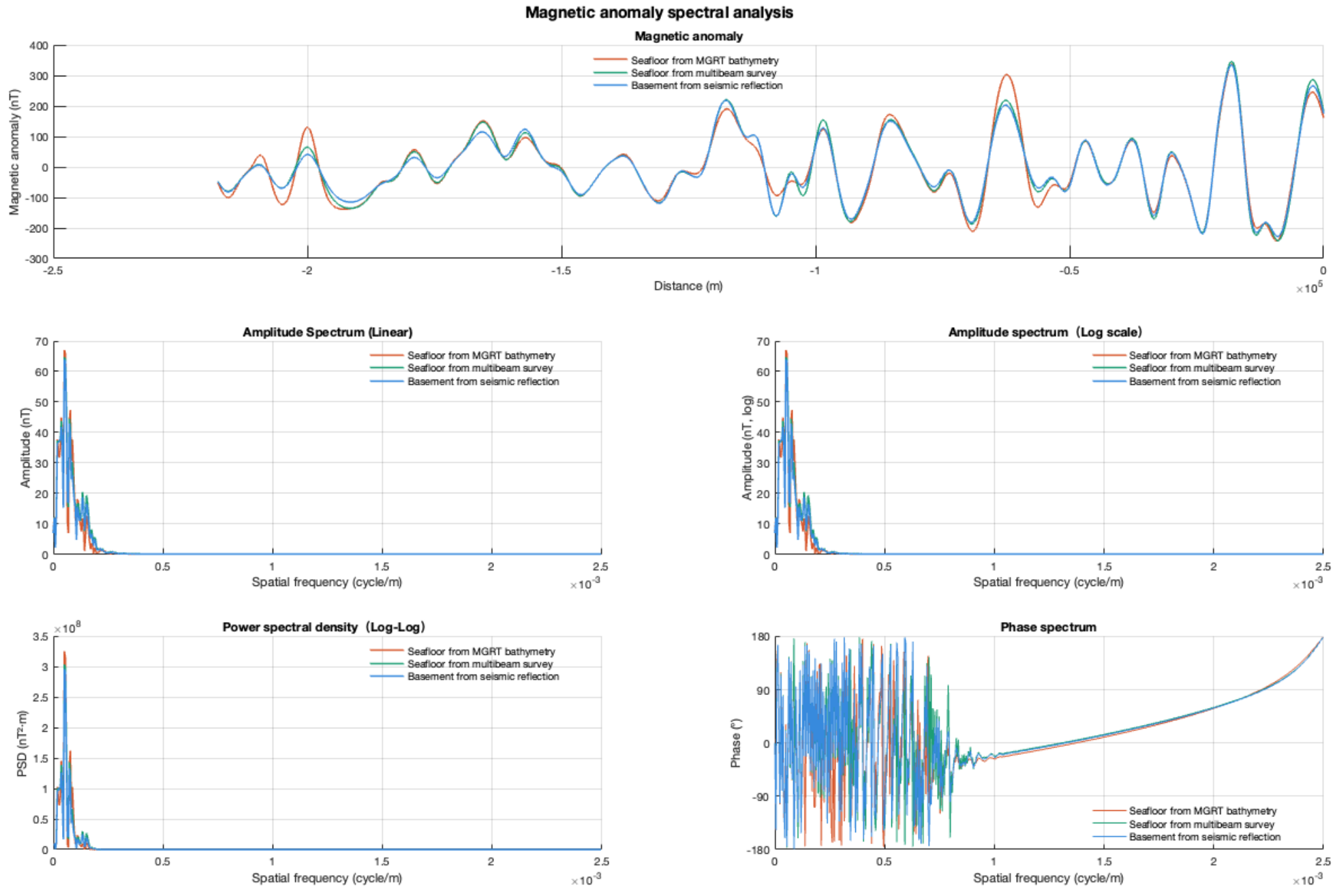

Figure 5. Spectral analysis of the magnetic anomalies calculated using different top boundaries of the magnetic layer.

Line 219: "randomly" is not very specific. Maybe you mean "uniformly randomly".

We mean that the melt bursts are not independent across magnetic profiles but are spatially and temporally correlated forming clusters. We reworded the sentence like this.

“Meanwhile, the melt bursts depicted by the magnetic profiles are not happening randomly and independently but seem to be spatially and temporally related and form clusters”

Thank you again for submitting your manuscript "Melt burst events in a dominantly amagmatic lithosphere: Gakkel Ridge, Arctic Ocean" to Nature Communications. We have now received reports from 2 reviewers and, based on their comments, we have decided to invite a revision of your work. Please address the remaining concerns raised by ref#1 (see their reports below).

Thank you for giving us the opportunity to improve the manuscript and reply to the reviewer. We also thank the reviewers' comments which are in black and our response in blue below.

REVIEWER COMMENTS

Reviewer #1 (Remarks to the Author):

The authors have made significant efforts to address the reviewer's comments, and the clarity of the manuscript has improved. However, I still feel that several issues remain and should be considered, together with a thorough polishing of the language and an improvement in figure quality.
The authors attempt to address the reviewer's two main concerns:
1. the uncertainty of the data relative to the analysed amplitudes, and
2. the need to demonstrate that introducing a patchy magnetization model is an alternative or superior approach compared to previous/standard models.

1. In Supplementary Figure S1, a comparison of two surveys is shown as a magnetic profile map. Three boxed areas are highlighted where the fit is considered particularly good. However, this is difficult to assess for several reasons: the two surveys are plotted using different colour schemes (the 1970s tracks in black and white, and the 1998–1999 data in blue and red), and the figure is too small to clearly evaluate the differences. A standard way to illustrate the quality of aeromagnetic data is to show the misfit at profile crossing points. This would allow us to assess both the magnitude of the errors and whether any spatial pattern exists in their distribution. In addition, total or regional RMS errors could be calculated and would provide useful information for the modelling.

We agree with the reviewer that a cross-over error analysis is the best way to assess the consistency of the two datasets. We proceeded with such an analysis. The crossing points are shown below (Fig. 1) and the mean difference between the two data sets at the crossing points is 11 nT, with a standard deviation of 78 nT. The RMS residual between the two data sets is 79 nT. This result indeed suggests that the two data sets are consistent. We added this analysis to the Supplementary Information. Furthermore, we still emphasize that adjacent profiles show basically the same features - only gradually changing from one domain (magmatic robust) to another (starved). As parallel lines were obtained at a different time, but show the same anomalies - the observed field must be well sampled.

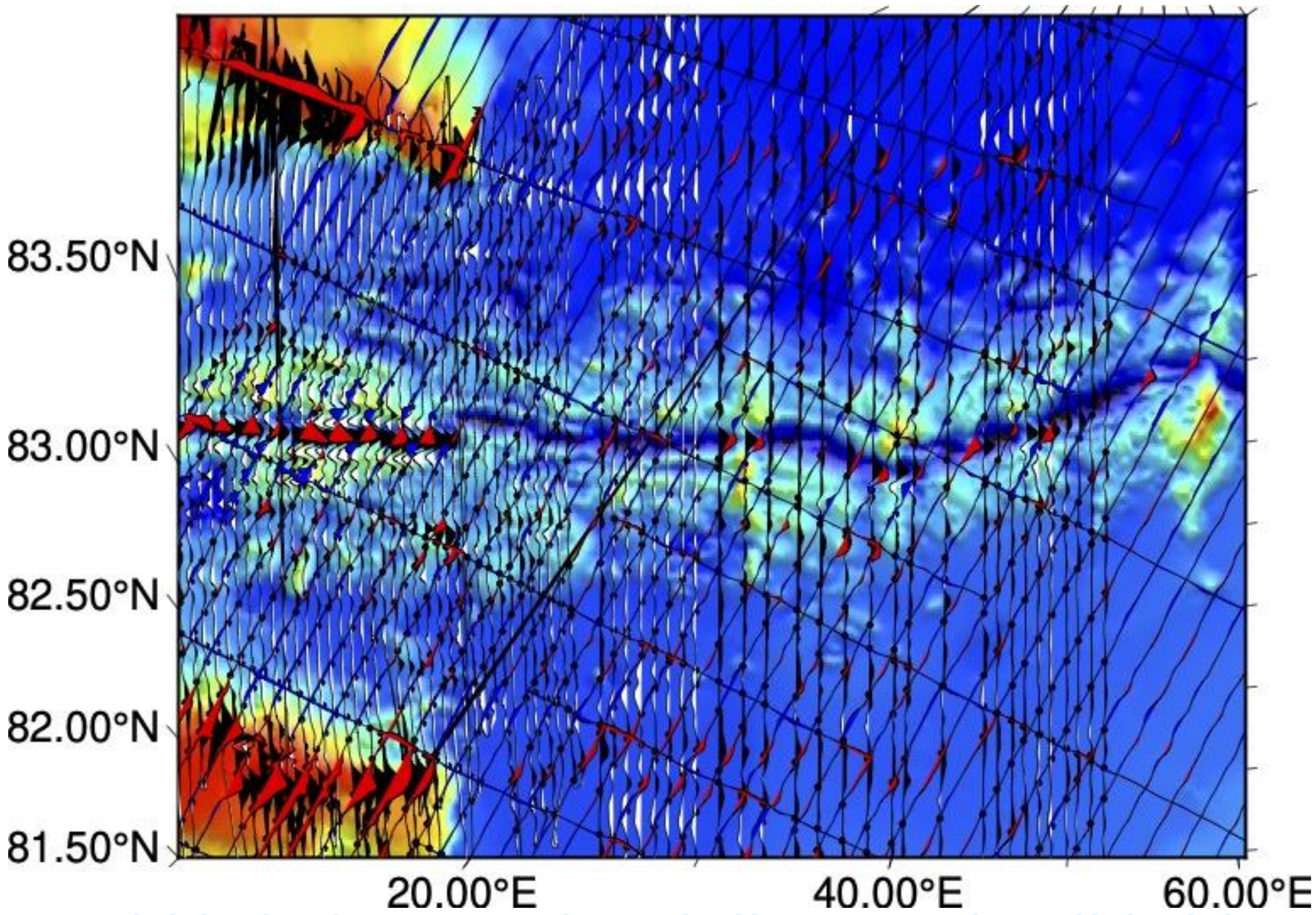

Figure 1: Black dots show the crossing points between the older aeromagnetic data set (black and white wiggles, tracks orthogonal to the spreading axis) and the new aeromagnetic data set (red and blue wiggles, oblique to the spreading axis).

2. I am still missing a figure comparing the full and patchy magnetization models. To me, the logical sequence would be to start from what was previously known or assumed and then demonstrate the improvement achieved in this study. For one or two representative profiles, show a model in which the top follows basement topography and the crust has a constant thickness of 2–4 km. Then apply a recent geomagnetic polarity timescale (e.g., Ogg 2020) and a spreading-rate history (e.g., Glebovsky et al., 2006), assuming magnetization throughout the entire crust. Compute the RMS error between the observed and modelled magnetic anomalies and analyse the distribution of the misfit. Next, use the same model geometry and spreading-rate history, but allow the magnetization to vary spatially and temporally according to the proposed patchy model. Again, compute the RMS error and analyse the misfit distribution.

Finally, discuss the strengths and weaknesses of both approaches and provide arguments supporting the preferred patchy saturation model.

We agree that the approach suggested by the reviewer is the logical one to study the magnetic anomalies of any oceanic basins, and indeed it was our first approach. But beyond the fact that it has been done by the previous studies and is therefore not original, this approach does not provide a good fit with the observed anomalies, requiring a more sophisticated approach in which only a part of the geomagnetic polarity sequence - during the production of magmatic crust - is actually making identifiable magnetic anomalies. To further substantiate this point (Fig. 2 below), we produced synthetic magnetic anomalies for the three real profiles shown in Figure 2 of the paper. On the right, we assume a constant-thickness magnetic layer, as suggested by the reviewer, and on the left we just duplicated the "patchy" approach shown in Figure 2 of the manuscript. The geomagnetic polarity timescale is the classic Cande and

Kent (1995) and the spreading rate was calculated from global plate motion calculator (University of Tokyo http://ofgs.aori.utokyo.ac.jp/~okino/platecalc_new.html) that integrates current existing NUVEL and MORVEL models. From top to bottom, the RMS residual between observed and synthetic anomalies are 277, 234, and 178 nT, respectively, for the constant thickness layer model and 105, 107, and 82 nT, respectively, for the "patchy" approach, i.e. two to three times larger under the assumption of a constant thickness layer, justifying our search for more suited models to fit the observations. The model with uniform magnetic layer may work well for the fast-spreading ridges with nearly constant crustal thickness, while the patchy model is more suitable for the ultraslow spreading ridges where the crust is often missing. We added this comparison and argument to the Supplementary Information Section 2.

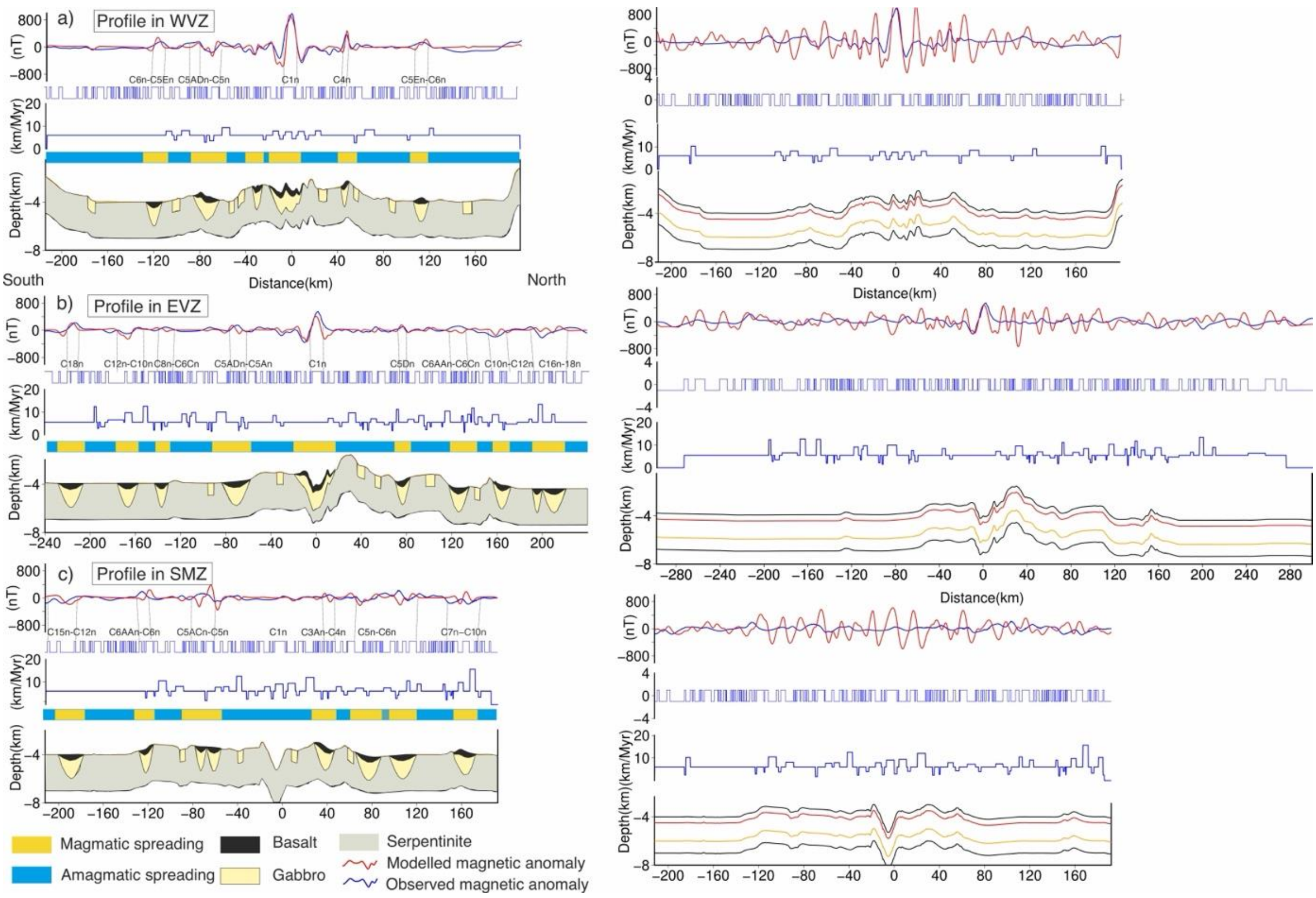

Figure 2: synthetic magnetic anomalies on representative profiles of the WVZ (top), EVZ (middle) and SMZ (bottom) sections assuming (left) our "patchy" approach duplicated from Figure 2 of the manuscript, and (right) a uniform thickness magnetic layer.

Other comments:

Figure S2: The polarity plot shows a strange “saw-tooth” pattern. Please correct this issue. The same problem appears in other figures. Is this a resolution issue?

This problem was just a drawing issue and has been fixed in the figures.

Figure S3: I find it difficult to believe that the global bathymetry is represented so poorly. Did you check the IBCAO grid? In the legend, both basement and topography are shown as red lines. In some places, the topography appears below the basement surface. Please check the resolution and plotting.

We plotted it again with the IBCAO grid (Fig. 3a) which has a better resolution in some areas. However, we insist that this did not alter the validity of our results, as shown by the modelling

below. On Fig. 3b, we investigate the effect of different topography assuming a constant thickness magnetic layer, with in green our previous bathymetric grid, in blue the IBCAO grid, in black the basement below the sediment given by a seismic profile, in red the topography along the same seismic profile (top panel). The effect on the magnetic anomalies (bottom panel) is negligible.

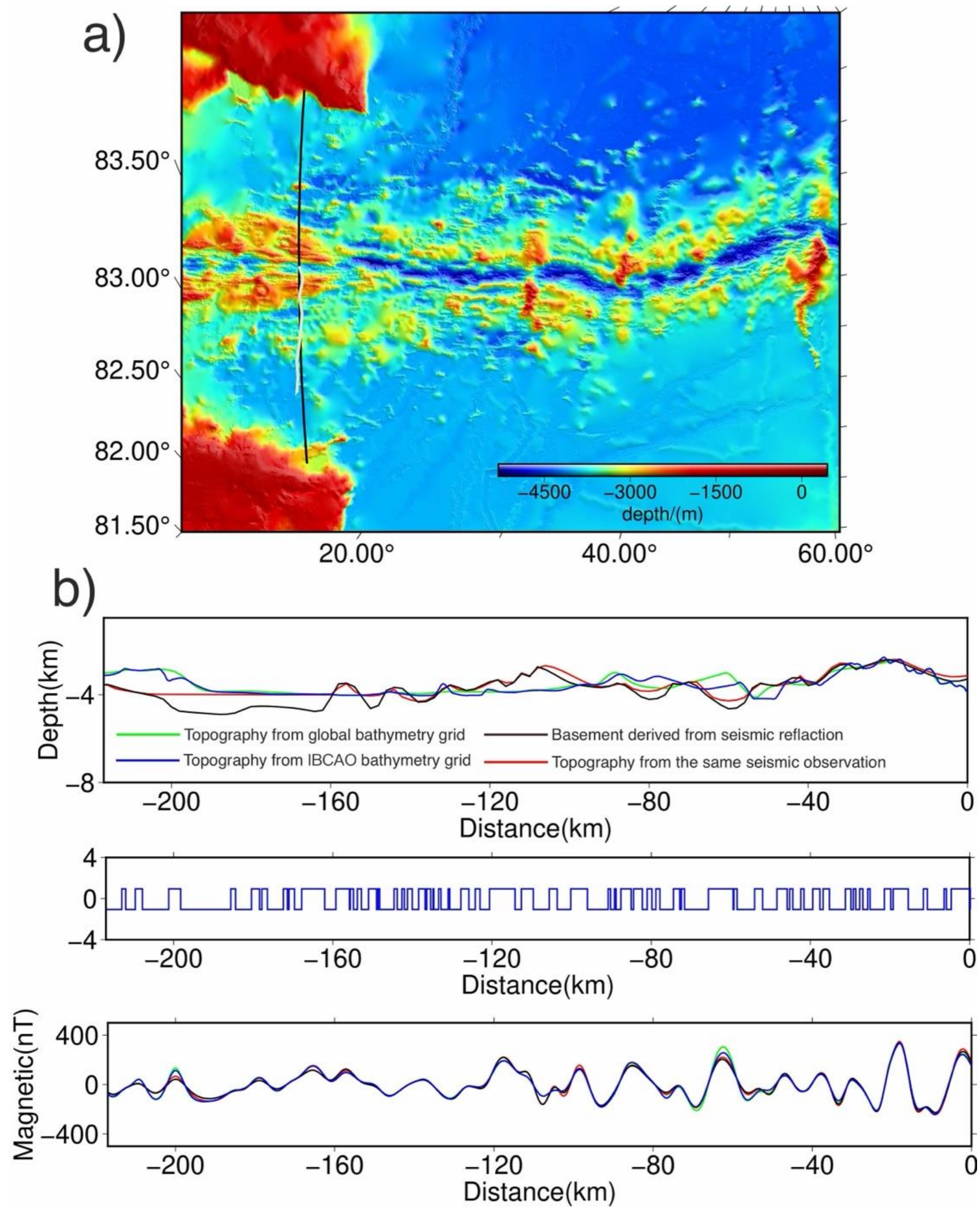

Figure 3: a): Bathymetry map from IBCAO grid. The black line marks the location of magnetic profile with the same location as a seismic survey (white line). b) Effect of different topography assuming a constant thickness magnetic layer, with in green our previous bathymetric grid, in blue the IBCAO grid, in black the basement below the sediment given by a seismic profile, in red the topography along the same seismic profile (top panel); magnetic anomalies (bottom panel)

Figure S4: Limit the x-axis to the range 0–0.5 cycles/m.
Modified accordingly

In the response, the isochrons modelled in this study are compared with grid contours. To me, the differences appear very large, especially for anomalies 6 and 13. The identified isochrons should instead be compared with the actual picks available from the same research group.
Interpreting magnetic anomalies at ultraslow spreading centers is a difficult exercise and it is not surprising that some interpretations may differ. To help the reviewer appreciate this difficulty, we have superimposed in Fig. 4 the interpretations of Gaina et al. (2002; picks obtained from Seton et al., 2014) and ours to the aeromagnetic grid and interpretation

REDACTED

Figure 4: Magnetic picks of Gaina et al. (2002; obtained from Seton et al., 2014; colored dots) and our isochrons (black lines) superposed to the aeromagnetic map and interpretation of Brozena et al. (2003). Anomalies 5, 6 and 13 are marked by yellow, orange and blue dots and the corresponding black lines.

presented by Brozena et al. (2003). The differences between the three interpretations are generally minor, except in a few limited corridors where the magnetic signal is very low or null. Consequently, these differences have no effect on our results.

Figure 1: Add chron C13 and provide the appropriate reference. Grid contours are not sufficiently accurate; actual picks should be used.

We have added C13 as suggested. The isochrons in this figure showing the "state of the art" before our study are from Müller et al. (2019) providing the new version grid of seafloor age, and the reference is now given in the caption. As the following Figure 5 shows, the magnetic picks obtained from Seton et al. (2014) and the grid contours from Müller et al. (2019) do not show significant differences, as the reviewer mentioned, they are related studies from the same research group. In addition, as mentioned above, some minor differences between different interpretations of the magnetic lineations are expected at ultraslow spreading ridges. Even the picks may be questionable in some areas by comparison to the actual magnetic wiggles.

Lines are easier to see than symbols, and there are already colored dots marking the lithology of dredges in the area. Plotting additional symbols may be confusing for the reader, and we therefore stand with lines.

REDACTED

Figure 5. Comparison of actual picks of Gaina et al. (2002; obtained from Seton et al., 2014; colored dots) and grid contours of the seafloor age from Müller et al., 2019 (black solid lines).

Figure 2: Correct the “saw-tooth” pattern, add a legend indicating the observed and modelled magnetic anomalies, and provide RMS errors. Also indicate the assumed mean spreading rate for each segment (in the figure or in captions), together with the mean and variance of the spreading-rate perturbations introduced in the model. Add a reference to the geomagnetic polarity timescale.

Saw-tooth pattern has been corrected and legend has been modified as suggested. The additional model suggested by the reviewer is shown together with the models from Figure 2 with the required RMS values. Reference of the magnetic polarity timescale, RMS errors,

assumed mean spreading rate and variance of the spreading rate perturbations introduced in the model have been added in the caption.

Reviewer #1 (Remarks on code availability):
Please, add a readme file, make sure that all dependencies/functions are included in the archive or provide a link where one can download them. Use relative paths.
A readme file has been included to introduce all the functions used for calculating the synthetic magnetic anomalies. Relative paths have been used. Links for downloading the data and codes in Data Availability and Code Availability have been updated.

Reviewer #2 (Remarks to the Author):

I find that the authors have satisfactorily addressed the comments in my first review, and I support publication in its present form.

John Goff
University of Texas Institute for Geophysics
